# A Hairpin Motif in the Amyloid-β Peptide Is Important for Formation of Disease-Related Oligomers

**DOI:** 10.1021/jacs.3c03980

**Published:** 2023-08-09

**Authors:** Mohammed Khaled, Isabel Rönnbäck, Leopold L. Ilag, Astrid Gräslund, Birgit Strodel, Nicklas Österlund

**Affiliations:** † Institute of Biological Information Processing: Structural Biochemistry (IBI-7), Forschungszentrum Jülich, 52428 Jülich, Germany; ‡ Department of Biochemistry and Biophysics, Stockholm University, 106 91 Stockholm, Sweden; § Department of Materials and Environmental Chemistry, Stockholm University, 106 91 Stockholm, Sweden; ∥ Institute of Theoretical and Computational Chemistry, Heinrich Heine University Düsseldorf, 40225 Düsseldorf, Germany; ⊥ Department of Microbiology, Tumor and Cell Biology, Karolinska Institutet − Biomedicum, 171 65 Solna, Sweden

## Abstract

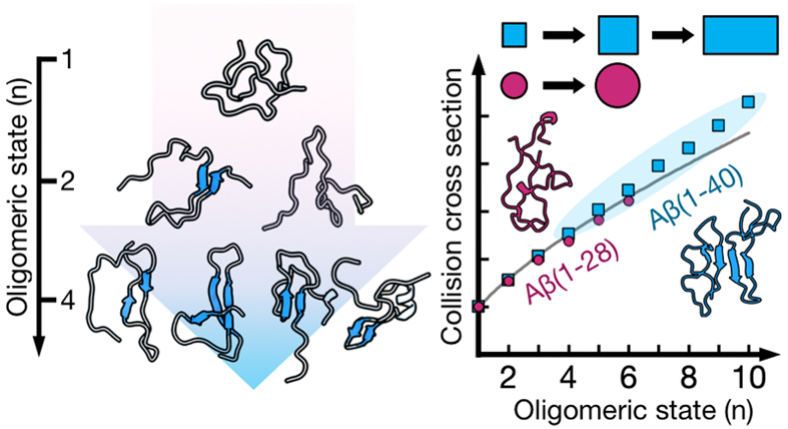

The amyloid-β (Aβ) peptide is associated with the development of Alzheimer’s disease and is known to form highly neurotoxic prefibrillar oligomeric aggregates, which are difficult to study due to their transient, low-abundance, and heterogeneous nature. To obtain high-resolution information about oligomer structure and dynamics as well as relative populations of assembly states, we here employ a combination of native ion mobility mass spectrometry and molecular dynamics simulations. We find that the formation of Aβ oligomers is dependent on the presence of a specific β-hairpin motif in the peptide sequence. Oligomers initially grow spherically but start to form extended linear aggregates at oligomeric states larger than those of the tetramer. The population of the extended oligomers could be notably increased by introducing an intramolecular disulfide bond, which prearranges the peptide in the hairpin conformation, thereby promoting oligomeric structures but preventing conversion into mature fibrils. Conversely, truncating one of the β-strand-forming segments of Aβ decreased the hairpin propensity of the peptide and thus decreased the oligomer population, removed the formation of extended oligomers entirely, and decreased the aggregation propensity of the peptide. We thus propose that the observed extended oligomer state is related to the formation of an antiparallel sheet state, which then nucleates into the amyloid state. These studies provide increased mechanistic understanding of the earliest steps in Aβ aggregation and suggest that inhibition of Aβ folding into the hairpin conformation could be a viable strategy for reducing the amount of toxic oligomers.

## Introduction

The small neuropeptide amyloid-β (Aβ) is highly aggregation-prone and spontaneously self-assembles in aqueous solution at concentrations higher than its equilibrium solubility.^1,2^ Intrinsically disordered Aβ monomers evolve during the aggregation process via several intermediate states into highly ordered and β-sheet-structured amyloid fibrils,^3^ which *in vivo* are deposited in neuritic plaques associated with Alzheimer’s disease (AD). The fact that Aβ peptides convert from an unstructured state into a β-sheet state means that aggregation occurs by nonclassical nucleation, as both aggregate size and β-sheet content must increase on the path toward nucleation.^4,5^ The so-called nucleated conformational conversion model describes how formation of amyloid nuclei occurs via structural rearrangement of some prenucleation aggregates.^6−8^ This structural conversion is associated with a high energy barrier, and primary nucleation is thus relatively slow.^9^ Structural templating using existing fibrils, so-called secondary nucleation, greatly reduces the activation barrier and is hence the dominating nucleation mechanism in Aβ aggregation.^10^

The precise mechanisms underlying the Aβ peptide’s toxicity and its connection to AD pathology are not yet fully understood. Prefibrillar aggregates have risen as especially interesting species in the aggregation process, as they are potentially more reactive than the mature and relatively chemically inert fibrils. The abundance of smaller aggregates has also been observed to correlate better with neurodegeneration compared to the mature fibrillar aggregates.^11,12^ Such prefibrillar aggregates range from dimers all the way up to large megadalton particles,^3,13−15^ with the smaller aggregates sometimes called oligomers (“oligo”, a few), while larger and more fibrillar-like aggregates are often termed protofibrils.^16^ However, no universally accepted terminology for different types of aggregates exists within the amyloid field. Prefibrillar aggregates have been the target for novel strategies to treat AD.^17,18^ Most recently, monoclonal antibodies that specifically target such aggregates have been developed, which are promising anti-AD drug candidates.^19−21^ Much is however still elusive about prefibrillar aggregates on the molecular level. The main reason for this is that they remain difficult to study due to their low abundance (the pool of soluble Aβ(1–42) oligomers never reaches more than 1.5% of the total peptide concentration in a buffered aqueous solution), low stability (oligomers are very dynamic and rapidly dissociate into monomers), and high polydispersity.^4^

**Figure 1:**
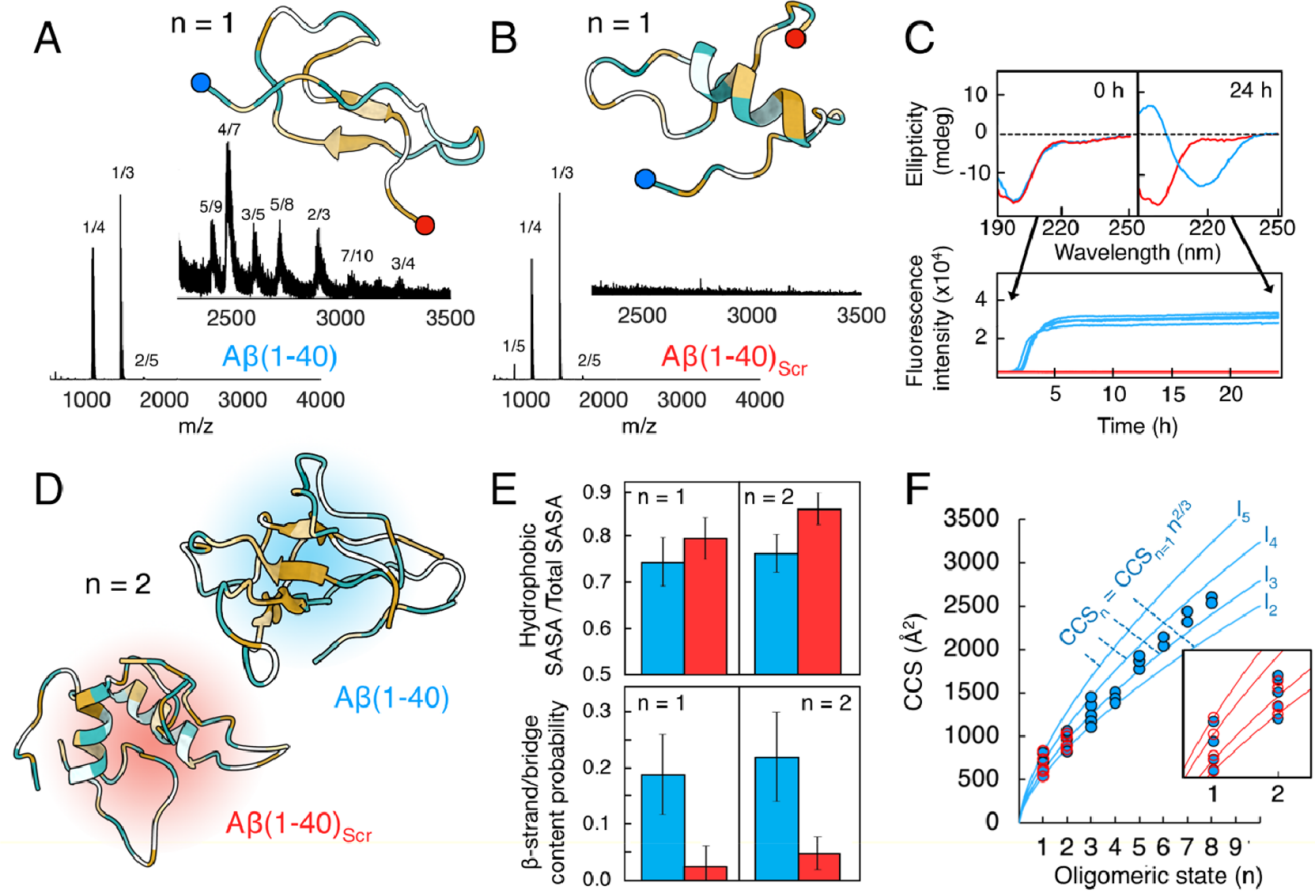
**Self-assembly of Aβ(1–40) and Aβ(1–40)**_**Scr**_**.** (A, B) Mass spectra of 20 μM (A) Aβ(1–40) and (B) Aβ(1–40)_Scr_ in 200 mM ammonium acetate (pH 6.8), with the oligomeric region magnified. Peaks are annotated by their oligomeric state/charge state ratio (*n*/*z*). The top cluster monomeric structure from 2 μs MD simulations is shown for each peptide, colored according to hydrophobicity (orange = hydrophobic, blue = hydrophilic). The N-terminus is highlighted in blue, while the C-terminus is highlighted in red. (C) Time-dependent aggregation assays on Aβ(1–40) (blue) and Aβ(1–40)_Scr_ (red) aggregation, as monitored by CD spectroscopy (top) and ThT fluorescence (bottom). (D) Top cluster structures from 10 μs MD simulations of Aβ(1–40) (blue) and Aβ(1–40)_Scr_ (red) dimers, colored according to hydrophobicity. (E) Calculated hydrophobic solvent-accessible surface area (SASA)/total SASA ratios (top) and β-strand/bridge content probabilities (bottom) from MD simulations for Aβ(1–40) (blue) and Aβ(1–40)_Scr_ (red) monomers and dimers. (F) Measured collision cross sections (CCSs) for each oligomeric state (blue circles for Aβ(1–40), red circles for Aβ(1–40)_Scr_). Solid lines represent the theoretical isotropic growth originating from each monomer state. The inset shows a magnification of the *n* = 1 and *n* = 2 oligomeric states.

The Aβ peptide sequence contains two hydrophobic segments, in the middle (the central hydrophobic core, CHC, residues 16–22) and in the most C-terminal region of the sequence (CTR, residues 30–42), separated by a relatively hydrophilic “hinge” segment (Figure S1). Proline scanning of the Aβ sequence has revealed that substitution in the hinge region increases the toxicity of the peptide, possibly due to stabilization of a turn motif that enables the two hydrophobic segments to interact more favorably.^22^ Phosphorylation of the S28 residue in the hinge segment has on the other hand been found to destabilize this turn motif due to repulsive electrostatic effects, which also leads to a loss in aggregation propensity.^23^ Transient back-folding of the C-terminal segment and formation of a transient β-hairpin structure (Figure 1A) are consequently believed to be important in Aβ aggregation and its associated disease-related toxicity.^24^ This is further supported by the fact that the hinge region is a segment where many disease-related mutations are located. Such disease-related variants include the Flemish (A21G), Dutch (E22Q), Italian (E22K), and Arctic (E22G) variants.^25−28^ The Arctic variant is particularly interesting, as this variant is enriched in toxic prefibrillar aggregates, which have been used as targets to develop the promising anti-AD antibody drug lecanemab.^29^ Stabilization of a partially folded hairpin state in Aβ has also been reported using an intramolecular disulfide bridge in the hinge region (A21C and A30C double mutant, Aβ_CC_). This restriction of conformational dynamics under oxidizing conditions similarly to the Arctic variant leads to the formation of stable and highly toxic prefibrillar oligomers, but no amyloid fibrils.^30^ These Aβ_CC_ structures have recently also been used to develop anti-AD antibodies to target especially neurotoxic Aβ species.^31^

In this study, the role of the seemingly very important β-hairpin motif in the earliest steps in Aβ peptide self-assembly is systematically examined biophysically. We use a combination of experimental *in vitro* techniques and computational modeling to study Aβ oligomers. Experimental studies of oligomers are generally challenging, as only a small fraction of the total peptide ensemble is in the oligomeric state at each given moment.^4^ One well-suited experimental technique for studying Aβ oligomerization is native mass spectrometry (MS), which provides highly resolved size information on the different coexisting assembly states in a heterogeneous mixture, even for sparsely populated states.^15,32^ Coupling of MS to ion mobility (IM) spectrometry furthermore provides low-resolution information about oligomer shape. Previous studies have compared experimental IM-MS data on Aβ oligomers to fibrillar Aβ structures,^14,33^ as no experimental atomistic structures of oligomers formed in simple aqueous solution are currently available. This is however problematic, as the oligomers represent a distinct state and are not just short fibrils.^4^

Another approach to studying the oligomeric ensemble and generating structure models is to conduct molecular dynamics (MD) simulations where monomeric peptides are allowed to form oligomers *in silico*. Such studies also provide challenges, as MD force fields have been originally developed for well-folded proteins and perform less reliably for disordered proteins.^34^ Many early MD studies of Aβ therefore tended to produce artificially compact conformations that do not accurately represent the intrinsically disordered nature of the peptide.^35^ We here use the Charmm36m force field, which has previously been determined to perform well for intrinsically disordered proteins (IDPs)^36^ and is currently the best for studies of protein aggregation.^37,38^ Simulation time is also critical, as IDPs sample a conformational space wider than that of folded proteins. We have therefore here run all-atom simulations of Aβ monomers, dimers, and tetramers totaling 56 μs to accurately capture the oligomerization process. Yet another challenge is to find a suitable starting structure, as most experimental structure models of Aβ monomers and oligomers have been determined in micellar environments or in organic cosolvents, which induce secondary structures not normally seen in pure aqueous solution.^39,40^ An alternative is the machine-learning-based structure prediction algorithm AlphaFold2, which has been shown to accurately identify disordered sequences.^41^ These static “structures” of IDPs do not provide any detailed information on the dynamic ensemble populated by such proteins^42^ but are here employed to generate starting structures for our MD simulations in cases where no suitable experimental structure is found in the Protein Data Bank.

The combination of IM-MS, machine-learning-based structure prediction, and MD simulations is very complementary: MD simulations provide high-resolution information on oligomer structure and dynamics, which is not obtainable by IM-MS. MS on the other hand reports on the relative populations of assembly states, which is challenging to obtain by MD because the entire energy landscape can rarely be sufficiently sampled.^9,43^ This combination of machine learning, MD, and native IM-MS to systematically examine different Aβ sequence variants here gives us valuable insights into the molecular mechanisms of Aβ aggregation and the nature of transient and low-abundance neurotoxic prefibrillar oligomers, which are highly interesting species in AD biology and drug development.

## Results

### Aβ Oligomerization Is Driven by Specific Structure Motifs

Mass spectrometry analysis of Aβ(1–40) results in detection of various oligomeric states at low relative abundance (Figure 1A), as has been previously reported.^14,15,44^ We here annotate the oligomers by their oligomeric state/charge state (*n*/*z*) ratio, as electrospray ionization (ESI) generates species with multiple charges. It should however be noted that oligomers can overlap in the *m*/*z* dimension of the mass spectrum, and for example, the *n*/*z* = 1/2 peak could hence consist of monomeric (+2), dimeric (+4), trimeric (6+), ... components. These components can, however, be deconvoluted using the ion mobility dimension, where ions are separated by a shape factor termed the collision cross section (CCS). There have previously been various reports regarding the oligomeric states populated by Aβ, with an early paper claiming that Aβ(1–40) only oligomerizes up to a tetrameric state.^44^ We here observe no such limitations, with Aβ(1–40) oligomers detected for at least up to an octameric state. There is also no specificity in which states are populated, with no preferred oligomeric state and a gradual decrease in intensity with increasing oligomeric state. This is in contrast to other more specific proteins detected by native MS^45^ as well as to Aβ(1–42) oligomers formed in the presence of zwitterionic detergent, where a preferred assembly state is formed.^40,46^ This indicates that Aβ oligomers in solution exist on a frustrated energy landscape, where no single aggregate state is significantly more favorable than the others.

Observation of oligomers in ESI-MS could theoretically be due to artificial clustering during the ionization process if two species are present in the same electrospray droplet upon desolvation. Low peptide concentrations and small droplet sizes (nanoESI) are used here to counteract this. Monte Carlo simulations have previously suggested that such nonspecific artifacts become relevant in nanoESI at protein concentrations greater than 50 μM,^47^ which is well above the concentration used here (20 μM).

To confirm that the detected oligomers are indeed specific aggregates, we analyzed a scrambled Aβ variant, Aβ(1–40)_Scr_, under conditions identical to those of wild-type Aβ(1–40). The Aβ(1–40)_Scr_ peptide has the same amino acid composition as Aβ(1–40), but hydrophobic residues are more evenly distributed over the sequence, in contrast to Aβ(1–40), where the hydrophobic residues are clustered in two segments (Figure S1). A scrambled peptide is a perfect control in a native MS experiment, as it has all the same average physicochemical properties as the original peptide, including the same mass. The mass spectrum for Aβ(1–40)_Scr_ shows a very similar charge state distribution as for Aβ(1–40) (Figure 1B), but Aβ(1–40)_Scr_ does not form any oligomers other than a dimer. The decrease in the aggregated forms of the scrambled peptide is seen also in the size exclusion chromatograms of the two peptides (Figure S2).

This observed difference in oligomerization propensity indicates that it is the unique sequence of Aβ(1–40) that drives its clustering into oligomers rather than a general phenomenon like the clustering of hydrophobic peptides in an aqueous environment or desolvation of multiple species from a single electrospray droplet. An explanation for the difference in the oligomerization propensity could be found by examining the structure propensities of the two peptides. Structure prediction using AlphaFold2 predicts that Aβ(1–40) forms a β-hairpin structure where the two hydrophobic segments self-interact (Figure S3A). The more even distribution of hydrophobicity in Aβ(1–40)_Scr_ is, however, predicted by AlphaFold2 to facilitate folding into an amphipathic helix (Figure S3B).

To test whether such structures could be stable in solution, we performed 2 μs MD simulations for each peptide monomer. While the helical motif in Aβ(1–40)_Scr_ remained during the MD simulation, most of the β-hairpin structure was not present in the top cluster MD model of the Aβ(1–40) monomer (Figure 1A, top). Circular dichroism (CD) spectroscopy confirms that fresh samples of both Aβ(1–40) and Aβ(1–40)_Scr_ are mostly unstructured, as shown by a spectral minimum at around 196 nm, which is characteristic for a random coil (Figure 1C, top). The Aβ(1–40) peptide, as expected, spontaneously evolves into large thioflavin T (ThT)-active aggregates upon incubation (Figure 1C, bottom, blue), which have a β-sheet structure, as shown by CD spectroscopy (Figure 1C, top, blue). In contrast, Aβ(1–40)_Scr_ does not form any such β-sheet amyloid aggregates upon incubation (Figure 1C, red).

Further 10 μs MD simulations were performed to study the differences between dimers formed by the two peptides. Aβ(1–40)_Scr_ was observed to interact within the dimer with the hydrophobic faces of the amphiphilic helices facing inward (Figure 1D). This structure, where hydrophobic patches have specific preferred interactions, could explain why Aβ(1–40)_Scr_ does not go on to form higher oligomers. Aβ(1–40) instead forms a dimeric structure by interpeptide interactions involving the β-strands in the most hydrophobic parts of the sequence (Figure 1D). Aβ(1–40) is found in the MD simulations to expose slightly fewer hydrophobic residues to the surrounding solvent compared with Aβ(1–40)_Scr_ (Figure 1E, top), highlighting that average hydrophobicity might not be the only driving force for oligomerization. Instead, the large difference in the β-sheet propensity (Figure 1E, bottom) and distribution of hydrophobic residues over the sequence could be important. It is easy to imagine how a β-sheet structure would enable continued growth of larger aggregates by addition of monomeric units without satisfying any particular aggregate number. This is demonstrated by modeling Aβ(1–40) tetramerization in MD simulations (also for 10 μs), where an even larger β-sheet core is formed (Figure S4A). Previous MD simulations of Aβ oligomers combined with transition network analysis have shown that several open and closed oligomeric structures of various aggregate sizes can be populated during further aggregation, in agreement with the mass spectrum in Figure 1A.^48^

The β-strand/bridge content of Aβ(1–40) increases in our MD simulations with increasing assembly state, from 19% in the monomer to 22% in the dimer and 30% in the tetramer (Figure S4B). Proline substitutions that reduce the aggregation propensity and neurotoxicity were also found to be in or close to the segments modeled as β-strands in our MD models of Aβ(1–40) (Figure S5A). Proline is a hydrophobic residue that acts as a β-sheet breaker. Loss of the β-hairpin motif in Aβ(1–40) is indeed suggested by AlphaFold2 when modeling the F19P variant (Figure S5B), which has previously been found to have decreased oligomerization propensity.^49^

### Small Aβ Oligomers Grow Isotropically

We next turned to ion mobility spectrometry to study the shapes of the Aβ(1–40) and Aβ(1–40)_Scr_ oligomers. All oligomeric states of the peptides display several conformations, as can be seen by the different collision cross sections (Figure 1F), which could represent either different solution-state structures or gas-phase structures. The experimental CCS values for Aβ(1–40) and Aβ(1–40)_Scr_ are similar for low *z* but are slightly increased for Aβ(1–40)_Scr_ at higher *z* (Figure 1F, inset). Calculated CCS values for the top clusters from MD simulations are also similar, with the Aβ(1–40) monomer having a CCS of 740 Å^2^ and the Aβ(1–40)_Scr_ monomer having a CCS of 780 Å^2^. These are within the experimentally measured range of values for monomers, which are between 530 Å^2^ (*z* = 2) and 830 Å^2^ (*z* = 5).

The experimental CCS values start to increase significantly above *z* = 3 for monomers and above *z* = 4 for dimers (Figure S6). High charge density in the gas phase will lead to strong Coulomb repulsion due to the low permittivity of vacuum. It can therefore be assumed that the compact ion mobility conformations observed at *z* = +2 and +3 for the monomer and +3 and +4 for the dimer are solution-state-like structures while the higher charge states which have significantly increased CCSs are structures that may have been significantly altered in the gas phase by repulsive intrachain Coulomb–Coulomb interactions. It can also be seen that most oligomers (with the oligomeric state *n*) observed have CCS values that can be fitted to the function CCS_*n*_ = CCS_*n*=1_ × *n*^2/3^, where CCS_*n*=1_ is the CCS of the compact *z* = +2 or +3 monomer. The implication of this is that the oligomers that follow this growth dependence grow as spheres, as the cross section of a growing sphere scales with the volume of the sphere raised to the power of 2/3, as previously described by Bleiholder et al.^50^ This agrees with the MD-generated structures, where oligomers with *n* = 1, 2, and 4 also appear to grow isotopically (Figure S7). We here annotate isotropic growth starting from the *n*/*z* = 1/2 and 1/3 states as I_2_ and I_3_, respectively (Figure 1F, blue solid lines). Higher oligomers above 6 deviate from these isotropic growth trends, indicating changes in aggregate shape.

### An Intramolecular Cross-Link in the Hinge Region Increases Oligomerization

**Figure 2:**
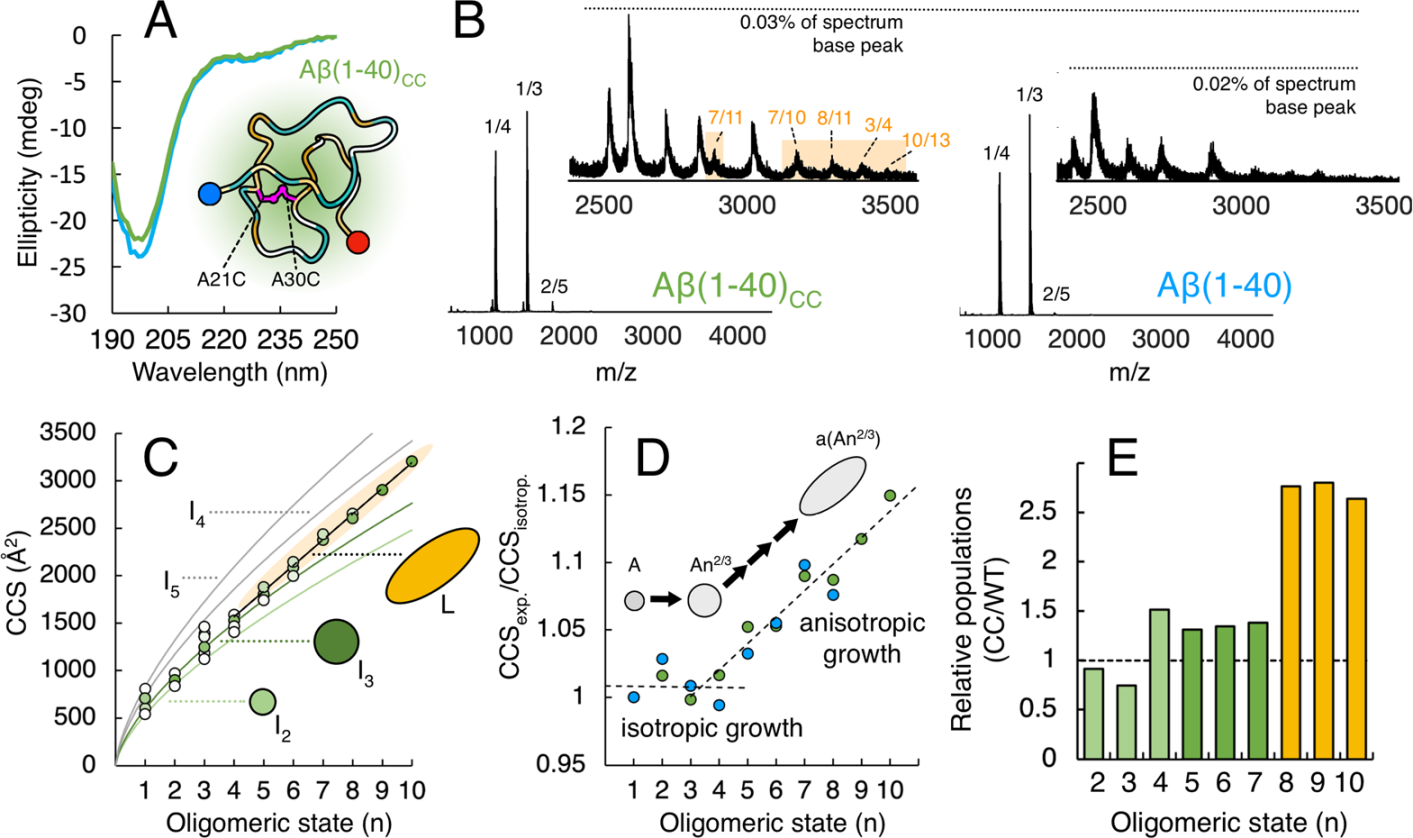
**Self-assembly of Aβ(1–40) and Aβ(1–40)**_**CC**_**.** (A) Far-UV CD spectra of 40 μM Aβ(1–40)_CC_ (green) and Aβ(1–40) (blue) in 20 mM phosphate buffer (pH 7.4), shown together with the top cluster structure after 2 μs MD simulations of the Aβ(1–40)_CC_ monomer, colored according to hydrophobicity (orange = hydrophobic, blue = hydrophilic). The N-terminus is highlighted in blue, while the C-terminus is highlighted in red. The disulfide bond formed between C21 and C30 is shown in magenta. (B) Mass spectra of 20 μM Aβ(1–40)_CC_ (left) and Aβ(1–40) (right) in 200 mM ammonium acetate (pH 6.8), with the oligomeric region magnified. Peaks are annotated by their oligomeric state/charge state ratio (*n*/*z*). States that are especially enriched in the CC variant are marked in orange. (C) CCSs of oligomers plotted against the oligomeric state. The experimental measurements are shown as circles colored according to relative intensity within an oligomeric state (white to green). Solid lines represent the theoretical growth behavior of isotropic growth (I) and linear growth (L). (D) Ratio between detected experimental CCS (intensity-weighted average) and the theoretical isotropic growth according to I_3_. The dashed lines represent fits to the data points between *n* = 1 and *n* = 4 and between *n* = 4 and *n* = 10. (E) Ratio between the relative intensity of oligomers in Aβ(1–40)_CC_ and in Aβ(1–40). Values above 1 indicate that the oligomeric state is increased in Aβ(1–40)_CC_ compared to that in Aβ(1–40).

As the sequence-specific β-hairpin structure motif seems to be important for the oligomerization of Aβ(1–40), we next attempted to stabilize this structure to see whether this would lead to an enhancement of the peptide’s oligomerization propensity. Stabilization of the β-hairpin state of the monomer has previously been explored by the introduction of an intramolecular disulfide bridge in the hinge segment (A21C, A30C).^30^ This double-mutant peptide, called Aβ_CC_, does not have a higher tendency to form amyloid fibrils—aggregation is instead halted at prefibrillar states. Aβ(1–40)_CC_ is coexpressed together with an affibody, which stabilizes the monomeric peptide in the β-hairpin conformation for which the structure has been solved using solution-state NMR spectroscopy.^30,51^ An MD simulation starting from an Aβ(1–40)_CC_ monomer in this hairpin state performed here for 2 μs suggests that the hairpin motif is not stable without the affibody, even though the two hydrophobic segments remain close to each other (Figure 2A). CD spectroscopy confirms this loss of β-sheet structure, as the Aβ(1–40)_CC_ variant displays a spectrum typical for a random coil, very similar to wild-type Aβ(1–40) (Figure 2A). Mass spectrometry analysis of Aβ(1–40)_CC_ reveals a general increase in oligomeric species compared to the wild-type variant, with a most notable increase for larger oligomers (*n* > 6) (Figure 2B, orange fields). Notable shifts in the ion mobility dimension are also seen, with shifts toward larger oligomers in peaks that overlap in the *m*/*z* dimension (Figure S8). This increase in oligomers for the Aβ_CC_ variant again points toward an important role of the hairpin structure in formation of oligomers.

The most populated charge states for each oligomeric state ≤4 follow isotropic growth that originates from the *z* = +3 monomeric state (I_3_). The I_3_ family extends from *n* = 1 to *n* = 6 but is not present for larger oligomers (Figure 2C, dark-green line). A less populated and more compact isotropic family I_2_ also exists that originates from the *z* = +2 monomeric state, which is populated between *n* = 1 and *n* = 4 (Figure 2C, light-green line). Above *n* = 4 the most populated charge states have CCSs that instead belong to a linearly growing family, L (Figure 2C, orange), that extends for as long as oligomers are detectable (at least *n* = 10). The ratio between the experimental population weighted average CCS for an oligomeric state and its theoretical isotropic I_3_ CCS for each oligomeric state clearly illustrates that the Aβ oligomers grow almost perfectly isotropic until *n* = 4, above which they start to deviate from isotropic growth (Figure 2D). Such a deviation indicates that the growth of the aggregates is faster in one specific direction, leading to the formation of elongated structures. Deviation from isotropic growth has also been shown to correlate with an increase in β-sheet content in studies of other amyloidogenic peptides.^52^ This could therefore indicate that monomers, dimers, and trimers are predominantly unstructured while oligomers >4 start to form more extended β-sheets. Comparison of the relative oligomer signals in the mass spectra for the wild-type and CC variants reveal differences between the oligomer distributions. Specifically, the CC variant exhibits a significant increase in oligomers with *n* ≥ 4, with a particularly pronounced effect observed for oligomers with *n* ≥ 8. Interestingly the CC variant has similar or slightly lower amounts of dimers and trimers compared to the wild-type (Figure 2E).

### Oligomer Growth Is Linked to Folding into the Hairpin Motif

**Figure 3:**
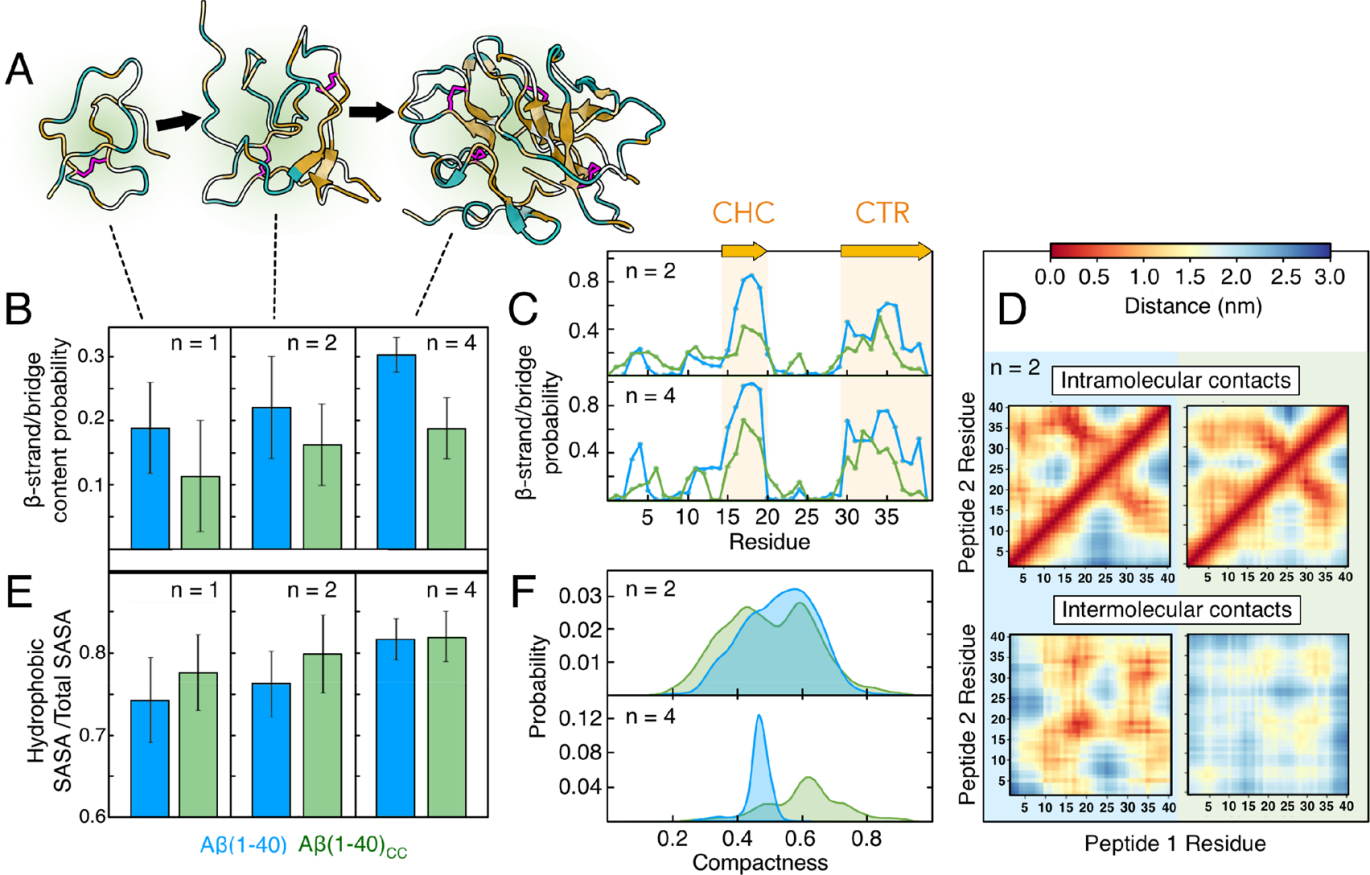
**Structural properties obtained by MD simulations for Aβ(1–40) and Aβ(1–40)**_**CC**_**.** (A) Most populated cluster structures of Aβ(1–40)_CC_ monomer, dimer, and tetramer, colored according to hydrophobicity (orange = hydrophobic, blue = hydrophilic). The clustering was performed by the gromos algorithm and a cutoff distance of 0.4 nm. (B) Average β-strand/bridge content probabilities for Aβ(1–40) (blue) and Aβ(1–40)_CC_ (green) for monomers, dimers, and tetramers. (C) Probabilities of β-strand/bridge of peptide residues for Aβ(1–40) (blue) and Aβ(1–40)_CC_ (green) for dimers (top) and tetramers (bottom). The two regions with high β-strand/bridge probability (CHC = central hydrophobic cluster, CTR = C-terminal region) are marked in yellow. (D) Intrapeptide (top) and interpeptide (bottom) contacts between residues for the Aβ(1–40) dimer (left, blue) and Aβ(1–40)_CC_ dimer (right, green). The intrapeptide contacts within peptide 1 are shown below the main diagonal and those within peptide 2 above the main diagonal. The color bar shows the average intra/inter-residue distance (in nm). (E) Ratios of the average hydrophobic solvent-accessible surface area (SASA) to the average total SASA for Aβ(1–40) (blue) and Aβ(1–40)_CC_ (green) for monomers, dimers, and tetramers. (F) Compactness distribution probabilities of Aβ(1–40) (blue) and Aβ(1–40)_CC_ (green) for dimers (top) and tetramers (bottom). The compactness values range from 0 (extended) to 1 (compact).

Native IM-MS suggests growth of oligomers, with a shift in structure occurring upon formation of larger assemblies. The technique is, however, not capable of determining the structure of the oligomers. For this, we instead analyze MD simulations of some of the species detected by mass spectrometry. We let the unstructured Aβ(1–40)_CC_ monomer assemble into dimers, which in turn assemble into tetramers (Figure 3A). The β-sheet propensity of Aβ(1–40)_CC_ increases when the oligomer size increases but is overall lower compared to the wild-type variant (Figure 3B). The increase in β-sheet propensity can also be seen in the top cluster structures (Figure 3A), as unstructured Aβ(1–40)_CC_ aggregates into a tetramer, where a core of short antiparallel β-sheets is formed by the most hydrophobic segment. Three of the four monomer units in the top cluster tetramer have folded into a hairpin structure similar to the β-hairpin found in the affibody complex, from which the starting monomeric structure was derived (Figure S9). It can be seen that the two hydrophobic segments (Figure S1) in the central hydrophobic core (CHC, residues 16–22) and the C-terminal hydrophobic region (CTR, residues 30–40) are indeed the segments with β-sheet propensity both in the wild type and the CC variant. These regions show β-sheet probabilities of around 60 to 90% in wild-type tetramers and around 40% to 70% in the CC variant tetramers (Figure 3C). For both peptides, the N-terminal region (residues 1–15) and the hinge region (residues 21–28), which links the β-strands, are highly disordered and tend to form β-bend/turn or random coil conformations (Figure S10). This agrees with experimental findings, although it is known that these segments could fold into helical structures at low temperatures.^53−55^ We do not see any such helical propensity within the wild-type oligomers in our simulations, which were performed close to the normal room temperature at 298 K. A small propensity to form helical structures is seen in Aβ(1–40)_CC_ oligomers (Figure S10).

The flexibility of the N-terminal region is also seen by analyzing the intramolecular contacts, where residues 5–10 make multiple short-range contacts with the entire peptide chain in both the dimer (Figure 3D, top) and the tetramer (Figure S11). The other significant interaction pattern is the intrapeptide interaction between the CHC and the CTR, which enables the formation of the hairpin motif. In Aβ(1–40)_CC_ the interaction between most C-terminal residues (35–40) and the CHC is reduced, which results in shorter β-sheets compared with the wild-type oligomers. The interpeptide contacts in the oligomers are also mostly between the hydrophobic segments with a high β-sheet propensity (Figure 3D, bottom). The interpeptide contacts are reduced in the Aβ(1–40)_CC_ dimers compared with the wild type. For the wild-type dimer, the highest contact density is observed between the two CHC regions, the regions with the highest β-sheet propensity (Figure 3C), which also display stronger interaction energies than interactions between the two CTR segments (Figure S12). The interaction energies reveal that the Lennard-Jones interactions within the wild-type dimer are stronger than those within the Aβ(1–40)_CC_ dimer. Similar patterns for interpeptide interactions are observed in the wild-type and CC tetramers (Figure S11). Overall, Aβ(1–40)_CC_ appears to be more disordered and expose slightly more hydrophobic surfaces than the wild-type variant, especially for the monomeric and dimeric states (Figures 3E and S10).

The flexibility of the peptides and their partial folding upon aggregation are further seen by analyzing the compactness of the oligomeric species (Figure 3F). Dimers of both peptide variants populate a wide distribution. The most populated state of the Aβ(1–40) dimer population has a compactness value of 0.6, while the Aβ(1–40)_CC_ dimer displays a more bimodal distribution with two peaks around 0.4 and 0.6. Aβ(1–40)_CC_ dimers display a wider distribution of compactness and also populate slightly more extended (less compact) states (Figure 3F, top). Further analysis also shows that this more extended Aβ(1–40)_CC_ state with a compactness value of around 0.4 has fewer intermolecular contacts and thus represents more open structures (Figure S13). The tetramers of both peptide variants are significantly less polydisperse, with sharper distributions (Figure 3F, bottom) compared to the dimers, with the Aβ(1–40)_CC_ tetramers adopting more compact states than the wild-type tetramers. This agrees with folding into more well-defined β-sheet structures upon oligomer growth.

### C-Terminal Truncation Leads to a Decrease in Oligomerization Propensity

**Figure 4:**
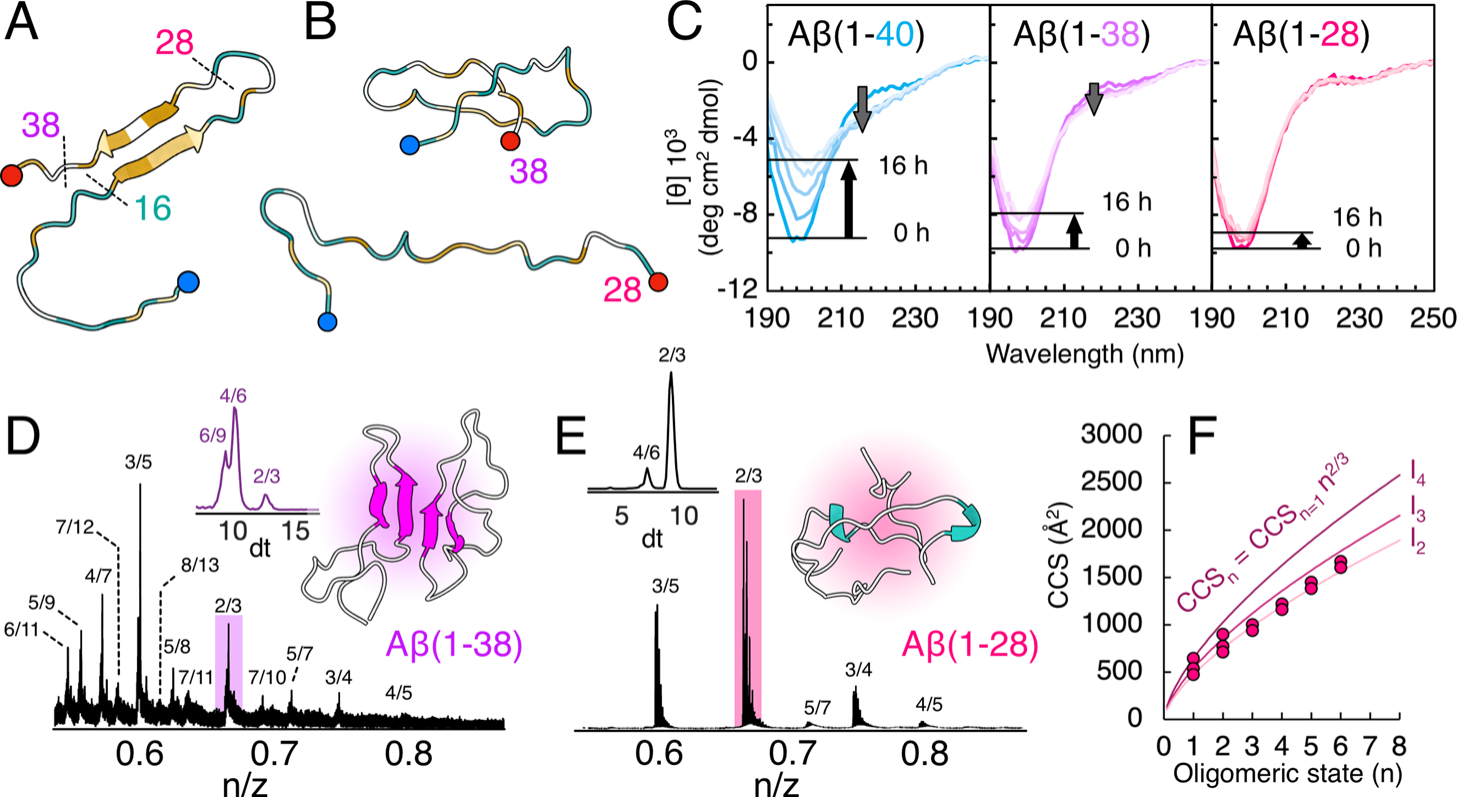
**C-terminally truncated Aβ variants.** (A) Overview of the different truncation variants. (B) Top cluster structures of Aβ(1–38) (top) and Aβ(1–28) (bottom) after 2 μs MD simulations, colored according to hydrophobicity (orange = hydrophobic, blue = hydrophilic). The N-termini are highlighted in blue, while the C-termini are highlighted in red. (C) Far-UV CD spectra of 40 μM Aβ(1–40) (blue), Aβ(1–38) (purple), and Aβ(1–28) (pink) in 20 mM phosphate buffer (pH 7.4). CD spectra recorded every 2 h for 16 h are shown. Arrows indicate changes at 196 (random coil signal) and 215 nm (β-sheet signal). (D, E) Oligomeric regions from the mass spectra of 20 μM Aβ(1–38) (D) and Aβ(1–28) (E) in 200 mM ammonium acetate (pH 6.8). Peaks are annotated by their oligomeric state/charge state ratio (*n*/*z*). Note that the *x* axes of the mass spectra are shown as oligomeric state (*n*)/charge state (*z*) (*m*/*z* divided by monomeric mass of the peptide variant) to enable easy comparison between the two variants. The ion mobility (drift time, dt) distribution for the *n*/*z* = 2/3 state (highlighted in a colored box) is shown as an inset for both variants. The top clusters of dimers of Aβ(1–38) (D) and Aβ(1–28) (E) are also shown as insets, colored according to secondary structure (coil = gray, sheet = magenta, helix = cyan). (F) Measured collision cross sections for each oligomeric state in Aβ(1–28), Solid lines represent the theoretical isotropic growth originating from each monomer state.

Our results indicate that folding of monomeric units into the β-hairpin structure is essential for the formation of larger Aβ oligomers, as they assemble by formation of extended antiparallel β-sheets. We next tested this by analyzing Aβ peptide variants which have been truncated from the C-terminal end, namely, Aβ(1–38), Aβ(1–28), and Aβ(1–16) (Figure 4A). As the hairpin is formed by interactions between the C-terminal and middle segments of Aβ, the truncation should lead to a lower propensity to fold into the hairpin structure and therefore a lower propensity to form higher oligomers. In Aβ(1–38), two hydrophobic valine residues, V39 and V40, are removed, which has previously been found to greatly reduce the amyloid aggregation rate.^56^ In Aβ(1–28) the entire CTR segment (_29_GAIIGLMVGGVV_40_) is removed, and the peptide thus only contains the N-terminal region, the CHC, and the hinge region. The N-terminal region in isolation, Aβ(1–16), was also analyzed.

An MD simulation of the Aβ(1–38) monomer shows that a compact hairpinlike state where the CHC and the C-terminal region are close to each other is still able to form, similar to that in Aβ(1–40) (Figures 4B, top and S14). MD simulations do, however, unsurprisingly show a loss of hairpin structure in Aβ(1–28) (Figure S14), as the entire second β-strand has been removed. The Aβ(1–28) monomer instead forms an entirely unstructured random coil (Figure 4B, bottom). CD spectrometry indicates that the truncated variants have random-coil-dominated spectral signatures, similar to the CD spectrum for Aβ(1–40). Truncation does, however, lead to a decrease in the aggregation propensity when incubated without agitation at 37 °C (Figure 4C).

Aβ(1–38) populates multiple oligomeric states (Figure 4D), similar to those populated by Aβ(1–40). This is in agreement with MD simulations of the Aβ(1–38) dimer that show formation of a similar antiparallel β-sheet structure as in Aβ(1–40). Truncation of the entire C-terminal region has a more drastic effect, with the oligomeric population of Aβ(1–28) shifted toward mostly dimeric and trimeric states (Figure 4E).

The *n*/*z* = 2/3 signal is especially highlighted to exemplify this shift (Figure 4D,E, colored boxes). This peak in the mass spectrum contains overlapping dimeric, tetrameric, and hexameric states in Aβ(1–38), with the tetramer being the main component. In Aβ(1–28) the distribution is heavily shifted toward the dimer, and the hexameric component is completely absent. Only minor pentameric (*n*/*z* = 5/7, 5/6) and hexameric (*n*/*z* = 3/4, 6/7) signals are overall seen for Aβ(1–28), with the hexamer being the highest oligomeric state detected in the spectrum. Ion mobility analysis shows that Aβ(1–38) oligomers follow similar growth trends as Aβ(1–40) (Figure S15). Aβ(1–28) on the other hand seems to only grow isotropically according to the most compact I_2_ family (Figure 4F). Aβ(1–28) forms a mostly unstructured dimer in MD simulations (Figure 4E), rather than β-strand interactions between the CHC regions in the two monomeric units as in Aβ(1–38) and Aβ(1–40) (Figure S16). The CHC is probably, however, involved in the formation of the observed small Aβ(1–28) oligomers, as the N-terminal segment Aβ(1–16) only forms monomers and dimers (Figure S17). Interactions between the CHC regions can indeed be seen in the intermolecular contact map for the Aβ(1–28) dimer, where peptide–peptide interactions are vague but clearly involve residues 15–20 (Figure S16).

## Discussion

### Formation of Oligomeric Structures

We have here exemplified, using both experimental and computational methods and by probing different Aβ variants, that a hairpin motif is important for the formation of oligomeric species. Especially the formation of larger oligomers (*n* ≥ 4) seems to require folding into the hairpin motif (Figure 2E). This hairpin motif arises due to the high β-strand propensity of two hydrophobic segments in the Aβ peptide and the transient folding of these segments onto each other. Such a β-hairpin structure enables the continued addition of monomeric units to form a larger β-sheet structure. The β-sheet propensity of Aβ increases with aggregation number (*n*), indicating some cooperativity in the folding process. This hairpin fold seems to also be stabilized by attractive electrostatic interactions between oppositely charged residues in addition to the hydrophobic interactions (Figure S12), with the importance of salt bridges being previously reported.^23,57^ This also points toward the possibility that association into oligomers could be heavily affected by pH and ionic strength.^58−63^ The exact effects on the here-observed oligomeric states when modulating the experimental conditions are currently unknown and warrant future studies.

The monomeric hairpin fold does not seem to be stable by itself in solution (Figure 2A), which is supported by earlier studies which employed solution-state NMR spectroscopy, CD spectroscopy, and IR spectroscopy.^54,58,64^ This highlights the intrisically disordered nature of the peptide, where multiple transient structures coexist on a rugged energy landscape, and the exact nature of the IDP ensemble will be highly dependent on experimental conditions.^53^ We do, however, find in our study that assembly of a larger β-sheet core makes the folding process more favorable and increases the stability of the structures. This could be compared to a “folding upon binding” event commonly observed for IDPs, with the unstructured Aβ monomer folding upon binding to a more structured Aβ oligomer. Interestingly, it is the folded hairpin motif suggested by AlphaFold2, which is not stable in the disordered monomer, that reappears in the oligomers.

A gradual increase in β-sheet structure has previously been observed by CD spectroscopy of isolated Aβ(1–40) oligomers stabilized by intermolecular photoinduced cross-linking of unmodified peptides (PICUP).^66^ Those PICUP-CD spectroscopy results report a β-sheet content of 24% in the monomer, which increases to 45% in the cross-linked tetramer. This can be compared to 19% for the monomer and 30% for the tetramer in our here-reported MD simulations.

**Figure 5:**
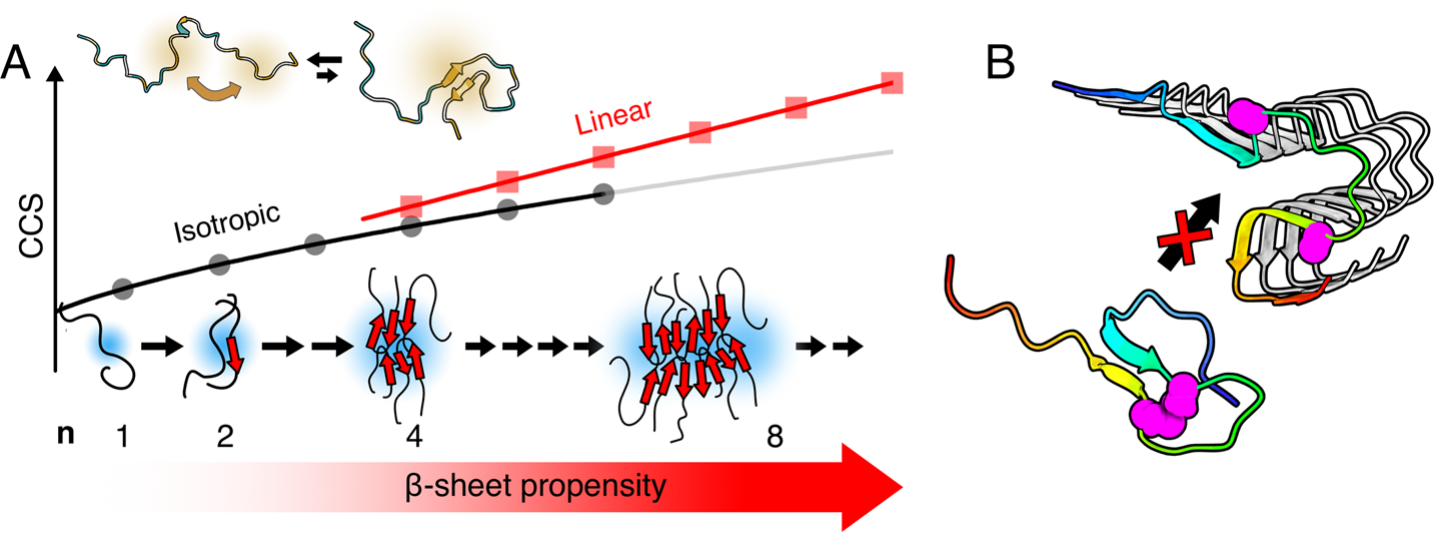
**Overview of the suggested steps in the early self-assembly process of Aβ peptides.** (A) Aβ peptides have a propensity to fold into a β-hairpin structure by interactions between the two hydrophobic segments in the peptide. Growth of Aβ oligomers follows isotropic growth until *n* = 4, at which point linear growth starts to dominate, indicating the formation of more elongated structures. A gradual increase of β-sheet content is also seen upon formation of larger oligomers in a “folding upon self-assembly” mechanism. (B) The structure in oligomers (generated by MD simulations) is distinct from the structure of fibrils (cryo-EM, PDB entry 7q4b(65)), and the formation of amyloid fibril requires a structural rearrangement. Residues 21 and 30, which are substituted for cysteines in Aβ_CC_, are shown as pink spheres.

An elongation in the IM structures occurs for oligomers larger than the tetramer. This could be interpreted as formation of an extended sheet structure (Figure 5A), as deviation from isotropic growth has been previously reported to correlate with an increase in β-sheet content in other amyloidogenic peptides.^52^ This illustrates that the gas-phase structures detected by IM-MS can also be used as proxy reporters on solution-state structures. It is furthermore seen in our study that the IM-MS results on oligomerization propensity agree well with the solution-state aggregation propensity of the peptide variants (Figures 1C and 4C).

It is also interesting to note that our previous study in membrane-mimicking micelles showed that all detected Aβ oligomers follow isotropic growth in such an environment and that oligomerization stops at the hexamer.^67^ We here observe that isotropic growth according to the I_3_ family also occurs up until the hexamer in a simple aqueous solution (Figures 1F and 2C). The conformation distribution for the hexamer is, however, heavily shifted toward the linearly growing oligomer family, which goes on to form even larger oligomers. This would suggest that the enrichment of oligomers in the micellar environment arises from stabilization of the globular-like (isotropic) Aβ structures by interactions with or within the micelle. This could, for example, be due to stabilization of hydrophobic surfaces and/or the increased electrostatic interaction strength within the low-permittivity, hydrophobic environment, which could increase the stability of the hairpin fold. This would inhibit continued growth into the extended and linearly growing oligomers observed in the absence of micelles. The micelles also inhibit formation of amyloid fibrils,^67,68^ indicating that the extended oligomers could be on pathway for fibril formation. The same reasoning holds true for the Aβ(1–28) peptide, which has a very low aggregation propensity and also does not form the extended oligomers (Figure 4F).

### Formation of Fibrillar Structures

The different oligomer conformations detected by ion mobility spectrometry were recently suggested by Lieblein et al. to correspond to different fibrillar morphologies.^14^ However, we consider this unlikely because oligomers are known to be structurally distinct from fibrils, as they for example are recognized by different antibodies.^69,70^ The difference between fibrils and oligomers has also been recently exemplified by fitting the growth of oligomers over time to kinetic models.^4^ Such models show that the occurrence of detectable metastable oligomers is not in agreement with direct formation of fibrils by elongation of oligomers as suggested by Lieblein et al.^14^ This is the case because elongation is energetically a very favorable process and would result in 5 orders of magnitude lower amounts of oligomers than are experimentally observed.^4^ Our MD simulations also give rise to Aβ peptides in distinct antiparallel β-sheet structures which have been classified as being characteristic of oligomeric Aβ aggregates.^40,71−73^ It is on the contrary known from solid-state NMR spectroscopy and cryo-electron microscopy that mature fibrils typically have parallel β-sheets.^65,74^

A structural rearrangement therefore needs to occur upon nucleation where the β-strands twist 90°, which involves breaking of intramolecular hydrogen bonds and salt bridge interactions within the hinge region and formation of new intermolecular hydrogen bonds (Figure 5B). Weakening of intramolecular ionic interactions which stabilize the hairpin state could therefore increase the rate of conversion into the fibrillar state. It has for example been reported that small Aβ aggregates formed at neutral pH are more toxic than larger rod-shaped aggregates formed at pH 5.^59^ It is also from this discussion obvious why the Aβ_CC_ peptides cannot form mature fibrils in their oxidized state, as such a rearrangement is not possible if residues 21 and 30 are covalently cross-linked together (Figure 5B, pink spheres). If the extended oligomers observed by ion mobility spectrometry were fibrillar-like, we would therefore expect them to be absent rather than increased in Aβ_CC_. Aβ_CC_ is instead known to form smaller aggregates which are also highly toxic.^30^

Oligomers with *n* = 12 (52 kDa) are not large complexes for native MS detection. It is therefore intriguing why we do not observe significantly larger oligomers in the mass spectra. One interesting and related question is at which point the Aβ oligomers interconvert into amyloid-like structures. Determining the critical size of the Aβ nucleus (*n*_c_) is difficult experimentally, as the nucleus represents a state with high free energy and is thus sparsely populated. However, modeling suggests that *n*_c_ occurs at relatively low *n*, typically between *n* = 6 and 14.^75−77^ The critical size has also been shown in such studies to depend on the peptide concentration^4,77^ and is also most likely also highly dependent on other solution conditions. A recent experimental study using fluorescence correlation spectroscopy showed that the BRICHOS chaperone, which inhibits fibril-dependent secondary nucleation, binds to Aβ aggregates as small as *n* = 8.^78^ This indicates that such small aggregates might already have converted to fibrillar-like aggregates, which can induce secondary nucleation. It is thus possible that the elongated oligomers that we observe in IM-MS are species that are able to convert into fibrillar states and quickly elongate. The propensity to convert should increase with increasing *n* according to modeling, but conversion is still a rare event.^4^ The inability of Aβ_CC_ oligomers to convert into the fibril state (Figure 5B) agrees with the observed increase of larger Aβ_CC_ oligomers.

### Relevance for Cellular Aβ Interactions

Early Aβ structures are believed to be especially toxic and therefore could be related to AD pathology. Our results show that oligomers are intrinsically polydisperse, indicating an underlying frustrated energy landscape. This is probably in part a reason for the toxicity of these species, as they are very prone to interact with other cellular species to reduce their chemical potential. Such cellular species could for example be the cellular membrane, resulting in a loss of cell or organelle integrity and uncontrolled leakage.^79−81^ The interaction with cell membranes will probably depend on the physiochemistry of the membrane, where certain lipids could stabilize or destabilize certain membrane-bound oligomeric states.^82^ Our earlier MS work has in fact shown that phosphatidylcholine lipids stabilize larger isotropic Aβ oligomers in micellar environments.^67^ Other works have also pointed toward the importance of anionic lipids, cholesterol, and gangliosides in Aβ–membrane interactions.^60,63,83−88^

The oligomers could also coaggregate with other biomolecules such as functionally important cellular proteins, resulting in breakdown of cellular function and loss of cellular proteostasis.^89,90^ Such events would under normal cellular conditions be prevented by control systems, such as chaperone proteins. It has interestingly been suggested that some chaperone proteins with antiamyloid activity, such as BRICHOS and the Hsp40-type DNAJB6 chaperone, might bind their clients by forming complementary β-strand/β-strand interactions between client and chaperone.^91^ DNAJB6 is known to specifically bind Aβ oligomers rather than monomers or fibrils and therefore must be able to discriminate between these structures.^92,93^ An NMR study has also shown that it is the hairpin-forming CHC and CTR segments in Aβ that become immobilized upon binding to Hsp104.^94^ All this could indicate that the transient β-hairpin motif is a structure that is recognized by important antiamyloid systems *in vivo*. Such specific binding would inhibit the formation of the frustrated extended oligomers which nucleate into amyloid.

The hairpin structure within Aβ, which is here shown to be of a transient nature in dilute *in vitro* samples, could hence be more commonly observed under crowded *in vivo* conditions due to interactions with various cellular interaction partners. It is interesting to note that AlphaFold2 might be able to pick up such conditionally folded structures, as it has been reported that IDP segments which are predicted with high pLDDT scores could in fact correspond to structures that form upon interactions with other proteins.^95^ Such interactions could also be with other copies of the Aβ peptide in the “folding upon binding” mechanism previously discussed.

### Relevance for Therapeutic Efforts

The findings presented here combined with observations from nature’s own antiamyloid systems, as described above, could provide useful insights into how to rationally design therapies against AD. Antibodies can be raised against epitopes of unknown structure, but other approaches would require more in-depth information about the molecular structures of the target species. It would be reasonable to imagine that inhibition of folding into the hairpin motif could be a possible strategy for preventing Aβ toxicity. This could be achieved by design of therapeutic peptides or proteins that bind with high affinity to the regions in Aβ with high β-sheet propensity and in that way outcompete the Aβ–Aβ interactions. The perhaps best example of this is antiamyloid peptides that use the Aβ(16–20) sequence (KLVFF) from the CHC.^96−100^ Our results clearly show how CHC–CHC interactions are important in oligomer formation (Figure 3D), meaning that this segment is indeed very promising to target to decrease the level of oligomer formation. Another approach would be to design therapeutic molecules that recognize and bind Aβ specifically in the hairpin motif, similar to the above-described chaperones. This could be achieved by designed small affinity proteins (so-called affibodies)^101,102^ or RNA aptamers.^103^

The integration of MD simulations and ion mobility mass spectrometry represents a powerful toolset for studying the elusive Aβ oligomers and exploring their modulation. By combining the rapid detection capabilities of IM-MS with the all-atom resolution of MD simulations, researchers can gain deep insights into the effects of molecules on oligomer populations and structures. Specifically, IM-MS enables efficient screening of many molecules by providing rapid detection of changes in relative oligomer populations.^104^ MD simulations can then be used to identify the structural effects of interesting molecules on the specific oligomer states observed in the experiments. This integrative approach has great potential to advance our understanding of the molecular mechanisms underlying Aβ oligomer formation and to help identify promising therapeutic targets for AD.

## Experimental Section

### Peptide Preparation

Lyophilized recombinant Aβ(1–40) was purchased from AlexoTech (Umeå, Sweden) (at a reported purity of >95% as shown by HPLC and SDS-PAGE). Lyophilized recombinant scrambled Aβ(1–40) (Aβ(1–40)_Scr_; Figure S1), Aβ(1–38), and Aβ(1–28) as well as lyophilized synthetic Aβ(1–16) were purchased from rPeptide (Watkinsville, GA, USA) (at a reported purity of >97% as shown by mass spectrometry). All peptides were dissolved in 6 M guanidine hydrochloride, purified, and buffer-exchanged into 200 mM ammonium acetate (pH 6.8) for IM-MS or 20 mM phosphate buffer (pH 7.4) for optical spectroscopy using either a Superdex Increase 75 10/300 or Superdex Increase 30 10/300 size exclusion column (Cytiva, Uppsala, Sweden). Only the peak corresponding to the monomer was collected. No unexpected sequence variants or unknown contaminations were observed in mass spectra of the size exclusion chromatography (SEC)-purified peptides, indicating a very high sample purity.

Recombinant Aβ(1–40)_CC_ peptides were kindly gifted by Professor Torleif Härd (SLU) and Professor Cecilia Emanuelsson (Lund University) and were provided as a coexpressed Aβ–affibody complex as described earlier.^30^ This Aβ–affibody complex was immobilized on a 5 mL HisTrap column (Cytiva). Aβ(1–40)_CC_ was separated from the immobilized affibody by washing with 20 mM phosphate buffer (pH 7.7), 150 mM NaCl, 10 mM imidazole, and 6 M guanidine hydrochloride. The isolated Aβ(1–40)_CC_ peptides were further purified and buffer-exchanged by using SEC as described above.

The concentrations of the obtained peptide fractions were determined by using UV absorption of the Y10 residue (ε = 1490 M^–1^ cm^–1^) residue by near-UV spectroscopy at 280 nm.

### Native Ion Mobility Mass Spectrometry

Native ESI-IM-MS was performed on a Synapt G2-S instrument equipped with an ion mobility cell. The peptides were diluted to a final concentration of 20 μM in 200 mM ammonium acetate buffer (pH 6.8). Samples were ionized in a nanoESI source using commercial metal-coated borosilicate spray emitters (Thermo Fisher Scientific). All peptides were ionized in the positive ion mode using a capillary voltage of 1.5 kV. The remaining settings were as follows: cone voltage of 50 V at an offset of 50 V, 25 °C source temperature, 10 mL/min trap gas. IM parameters were set at a wave height of 40 V and a wave velocity of 1200 m/s. The collision energy in the ion mobility cell was set at 5 V.

Drift time information from each measurement was retained using DriftScope (Waters, Milford, MA, USA). Drift times from IM-MS for peptides were calibrated to obtain CCS values as previously described.^105^ Bovine ubiquitin (Sigma-Aldrich), bovine β-lactoglobulin (Sigma-Aldrich), and honeybee melittin (Sigma-Aldrich) were used to create the CCS calibration curve, as these proteins span the size scale of Aβ oligomers. The reference CCS values for the calibrant proteins were obtained from the literature.^45,106^ The obtained raw data from mass spectrometry were analyzed using the software massLynx v4.1 (Waters).

The growth of collision cross sections was fitted to previously derived growth equations.^50^ Spherical growth is described by the equation CCS_*n*_ = CCS_*n*=1_ × *n*^2/3^, as CCS_*n*_ = π*r*_*n*_^2^ = π[(3/4π)*V*_*n*_]^2/3^ and *V*_*n*_ = *nV*_*n*=1_ according to spherical geometry,^50^ where *n* is the oligomeric state, CCS is the cross section of the sphere, and *V* is the volume of the sphere.

### Circular Dichroism Spectroscopy

CD spectrometry was performed on a Chirascan spectrometer (Applied Photophysics, Leatherhead, U.K.) using a 2 mm quartz cuvette. The ellipticity between 250 and 190 nm (1 nm step size, 4 s sampling time per point) was measured on samples of 40 μM peptide in 20 mM phosphate buffer (pH 7.4). The secondary structures of Aβ peptides were studied over time in aggregation kinetics experiments for a total of 18 h at 37 °C without agitation.

### Protein Structure Prediction

All predictions were generated using the online version AlphaFold2 w/MMseqs2, no templates, within ColabFold.^107^ The predicted local-distance difference test (pLDDT) score was used to evaluate the prediction accuracy. A pLDDT value < 50 indicates poor prediction and pLDDT > 90 corresponds to high confidence.^107,108^

### Molecular Dynamics Simulations

The simulations of the wild-type Aβ(1–40) monomer were initiated from a β-hairpin conformation (PDB entry 2otk(30)). The mutated Aβ(1–40)_CC_ with the disulfide bond was built from the same structure by mutating the wild-type Aβ(1–40) amino acids A21 and A30 to C21 and C30 using Charmm-GUI,^109^ and to mimic the experimental conditions, a methionine amino acid was added to the N-terminus using PyMOL (Schrödinger)^110^ and the ModLoop server^111^ to relax the N-terminal residues. The initial structures of Aβ(1–40)_Scr_, Aβ(1–38), and Aβ(1–28) peptides were predicted by AlphaFold2.^107^

The initial structures were placed in a cubic box; water molecules and 150 mM NaCl were added, and the systems were neutralized by inserting extra Na^+^ ions. After that, the systems underwent energy minimization using the steepest descent algorithm to remove clashes between atoms,^112^ followed by two equilibration steps, each for 1 ns, under canonical (*NVT*) and isobaric–isothermic (*NPT*) ensemble conditions. During the equilibration, the pressure was kept at 1.0 bar using the Parrinello–Rahman pressure coupling method,^113,114^ and the temperature was kept at 298 K using the velocity-rescale thermostat method.^115^ The production MD simulations were conducted under the *NPT* ensemble conditions. Electrostatic interactions were calculated using the particle mesh Ewald method with a real-space cutoff distance of 1.2 nm; van der Waals interactions were also cut off at 1.2 nm.^116^

Each monomer was simulated for 2 μs. For the dimer systems, two monomers obtained from the highest populated clusters were added to the simulation box 0.8 nm away from each other to initiate the dimer simulations. The dimers were simulated for 10 μs for Aβ(1–40) and Aβ(1–40)_CC_ and 2 μs for Aβ(1–40)_Scr_, Aβ(1–38), and Aβ(1–28). Moreover, in the cases of Aβ(1–40) and Aβ(1–40)_CC_, tetramers were also simulated, each for 10 μs, starting from the highest populated dimer structures placed 0.8 nm away from each other. The same simulation setups as used in the monomer simulations were used for the dimer and tetramer simulations.

All MD simulations were performed using GROMACS 2020/2022^117,118^ with the Charmm36m force field^36^ and the TIP3P water model.

## Supporting Information Available

Hydrophobicity profiles for Aβ(1–40) and Aβ(1–40)_Scr_ (Figure S1); size exclusion chromatograms for Aβ(1–40) and Aβ(1–40)_Scr_ samples (Figure S2); predicted AlphaFold2 models for Aβ(1–40) and Aβ(1–40)_Scr_ (Figure S3); structural development of Aβ(1–40) monomer and oligomers during MD simulations (Figure S4); structure models of Aβ(1–40) and the effect of proline substitutions (Figure S5); CCS values for Aβ(1–40) and Aβ(1–40)_Scr_ monomers and dimers as a function of charge state (Figure S6); comparison of theoretical and experimental CCS values for Aβ(1–40) (Figure S7); example experimental drift time profiles for Aβ(1–40) and Aβ(1–40)_CC_ (Figure S8); structures of the monomeric units of the Aβ(1–40)_CC_ in the top clusters of oligomers from MD simulations (Figure S9); probability of secondary structure of the residues of Aβ(1–40) and Aβ(1–40)_CC_ (Figure S10); average intra- and interpeptide contacts for Aβ(1–40) and Aβ(1–40)_CC_ tetramers (Figure S11); average interpeptide interaction energies between residues for Aβ(1–40) and Aβ(1–40)_CC_ dimers (Figure S12); cluster analysis of Aβ(1–40) and Aβ(1–40)_CC_ with calculated free energy surfaces (Figure S13); β-strand/bridge content probability for Aβ(1–40), Aβ(1–38), Aβ(1–28) monomers and dimers (Figure S14); measured CCS values for Aβ(1–38) oligomers (Figure S15); average intra- and interpeptide contacts for Aβ(1–38) and Aβ(1–28) dimers (Figure S16); and mass spectrum of Aβ(1–16) (Figure S17) (PDF)
