# Supplementary material for: A Hairpin Motif in the Amyloid-β Peptide Is Important for Formation of Disease-Related Oligomers
Source: J Am Chem Soc. 2023 Aug 9;145(33):18340–54. doi: 10.1021/jacs.3c03980 (PMC10450692; doi:10.1021/jacs.3c03980)
Supplement: Supplementary file 1 — ja3c03980_si_001.pdf [file ja3c03980_si_001.pdf]

Supporting information for

A hairpin motif in the Amyloid-β peptide is important for formation of disease-related oligomers

Mohammed Khaled[1], Isabel Rönnbäck[2], Leopold L. Ilag[3], Astrid Gräslund[2], Birgit Strodel[1,4*], Nicklas Österlund[2,3,5*]

1. Institute of Biological Information Processing: Structural Biochemistry (IBI-7), Forschungszentrum Jülich, 52428 Jülich, Germany.
2. Department of Biochemistry and Biophysics, Stockholm University, SE-114 18 Stockholm, Sweden.
3. Department of Materials and Environmental Chemistry, Stockholm University, SE-114 18 Stockholm, Sweden.
4. Institute of Theoretical and Computational Chemistry, Heinrich Heine University Düsseldorf, 40225 Düsseldorf, Germany.
5. Department of Microbiology, Tumor and Cell Biology, Karolinska Institutet – Biomedicum, 171 65 Solna, Sweden.

Thid pdf includes:
Supporting Figures S1-S18
References for the Supporting Information

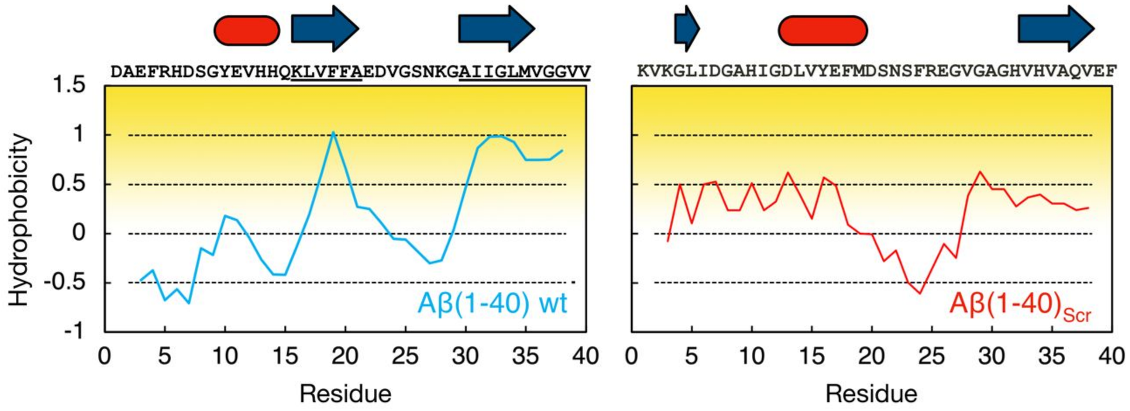

Figure S1. Overview of peptide sequences, Aβ(1-40) (left) and Aβ(1-40)$_{Scr}$ (right). Structure predictions by jpred (1)are shown on the top. Sheets are represented by blue arrows, helices by red ovals. Calculated hydrophobicity by the Eisenberg method is plotted against the sequence, using an averaging window of 5 residues.

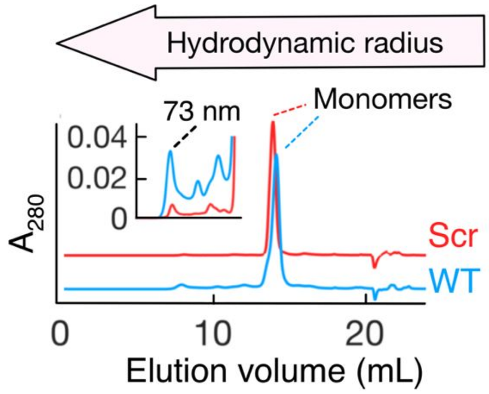

Figure S2. Size exclusion chromatography of Aβ(1-40) (blue) and Aβ(1-40)$_{Scr}$ (red) performed on a Superdex 75 10/300 Increase (Cytiva) column. The insert shows a magnification of aggregates in the chromatogram.

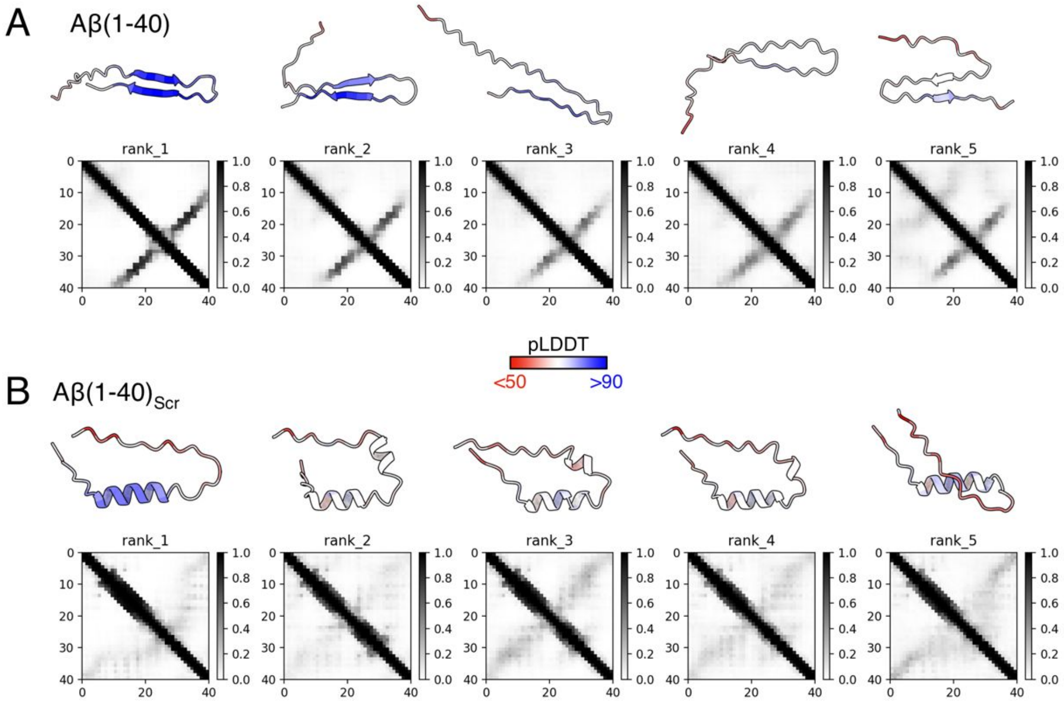

Figure S3. Predicted AlphaFold2 Models for **A)** Aβ(1-40) and **B)** Aβ(1-40)$_{Scr}$. The generated models are colored according to their pLDDT-score (red to blue). Scores >90 (blue) are expected to be modeled with high accuracy. Scores between 70-90 (white to blue) are expected to be modeled well. Intramolecular contact maps are shown below each structure model.

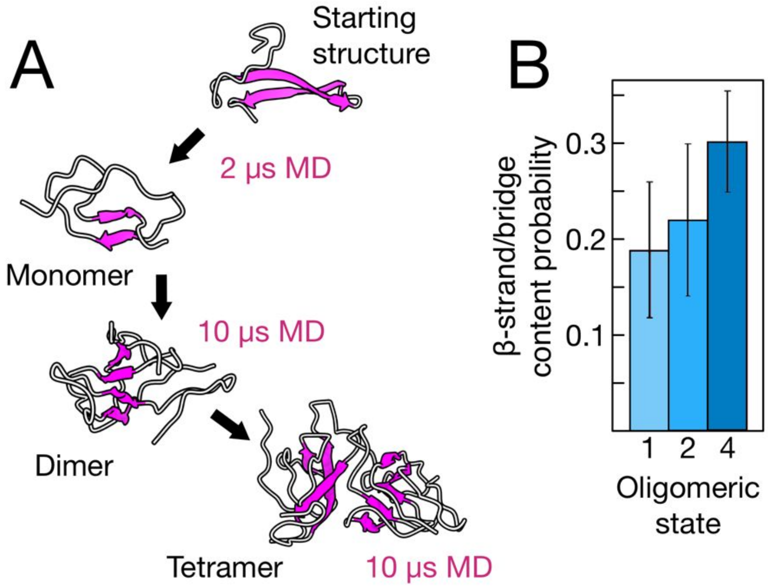

Figure S4 A) Structural development of Aβ(1-40) monomer and oligomers in MD simulations. The starting structure is taken from the NMR-derived structure of Aβ(1-40) in an affibody complex (pdb 2otk). Sheets are colored magenta. **B)** β-strand/bridge propensity (average plus standard deviation) for Aβ(1-40) species calculated from the MD simulations.

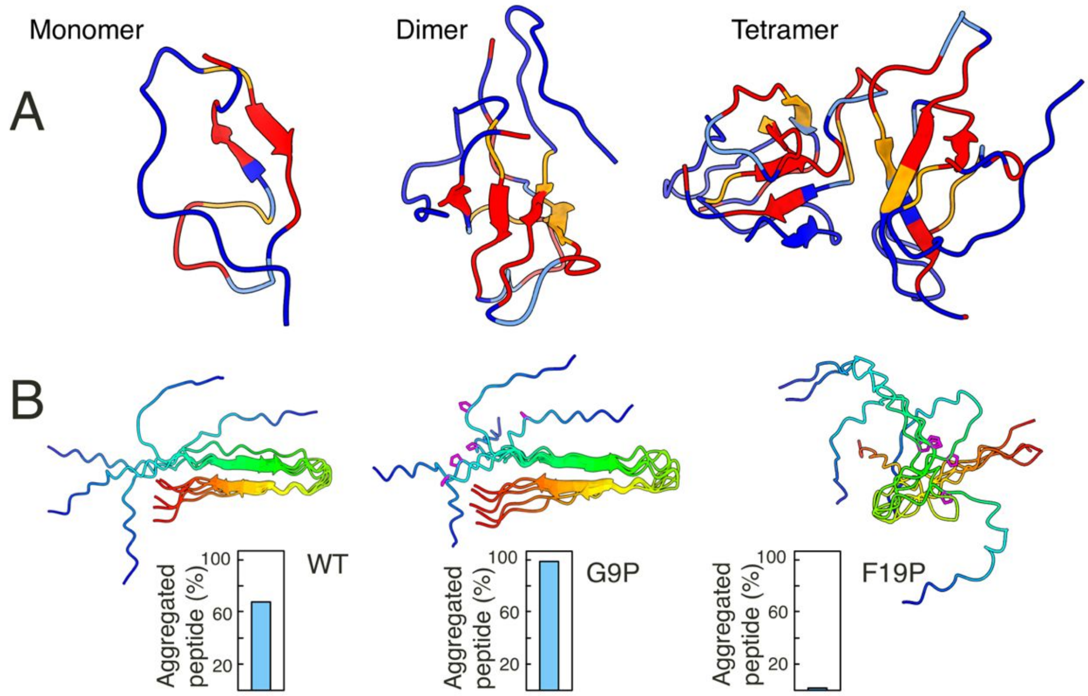

Figure S5. A) Top cluster structures from MD simulations of Aβ(1-40) colored according to the effect of proline substitutions in reference (2) (orange-red represents inhibition of aggregation, blue represents no effect on aggregation). **B)** AlpaFold2 models of wild-type Aβ(1-40) and two proline substitutions. The G9P substitution does not affect the hairpin fold and does not inhibit aggregation. The F19P substitution breaks the hairpin and inhibits aggregation. Data on aggregation propensity was collected from reference (2)

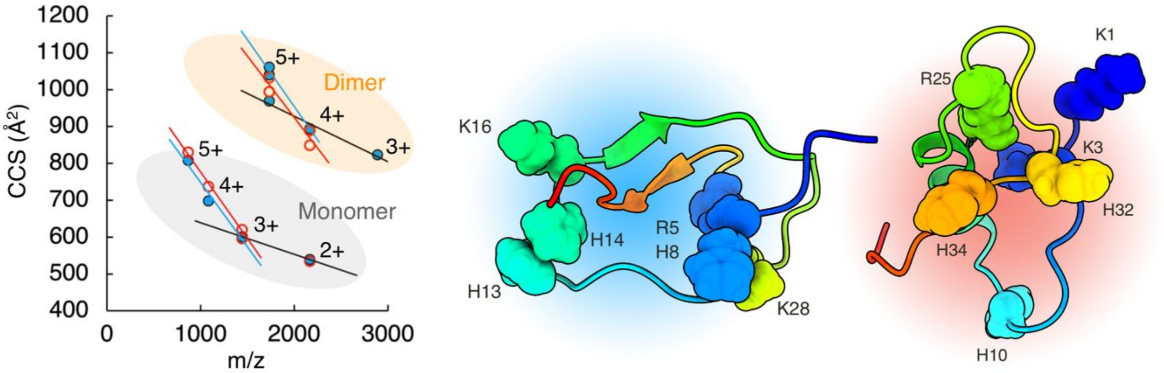

Figure S6. (left) Experimental CCS values of Aβ(1-40) (blue) and Aβ(1-40)$_{Scr}$ (red) for different charge states. Solid lines represent linear fits to the experimental data. Higher charge states display a steep increase in CCS with charge state, likely due to intrachain repulsion events in the gas phase. **(right)** Positively charged residues in the two peptide variants are shown as spheres in the MD derived structures.

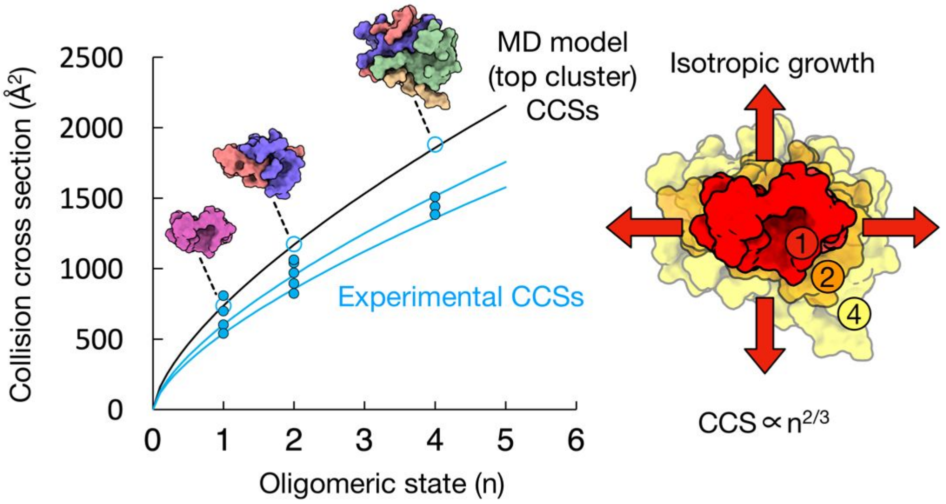

Figure S7. Calculated CCS values for the top clusters of Aβ(1-40) generated from MD simulations are shown as open blue circles. The theoretical isotropic growth originating from the MD monomer is shown as a black solid line, indicating that the MD models grow isotopically (spherically) with respect to oligomeric state. Experimental CCS values are shown in solid blue circles for comparison.

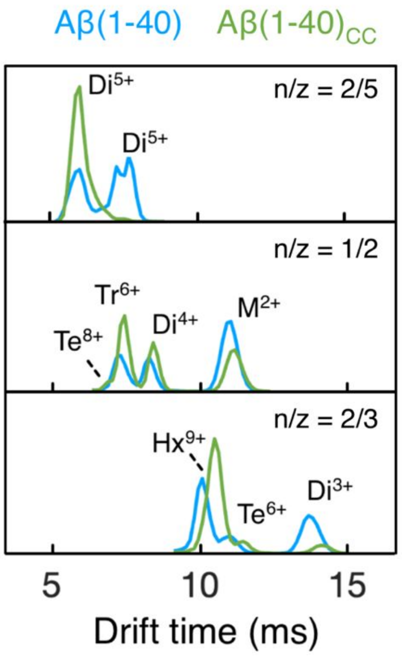

Figure S8. Example experimental drift time profiles for some n/z signals from Aβ(1-40) (blue) and Aβ(1-40)$_{CC}$ (green). The profiles are normalized with respect to their total signal intensity to compare the relative abundances within each n/z signal between the two variants.

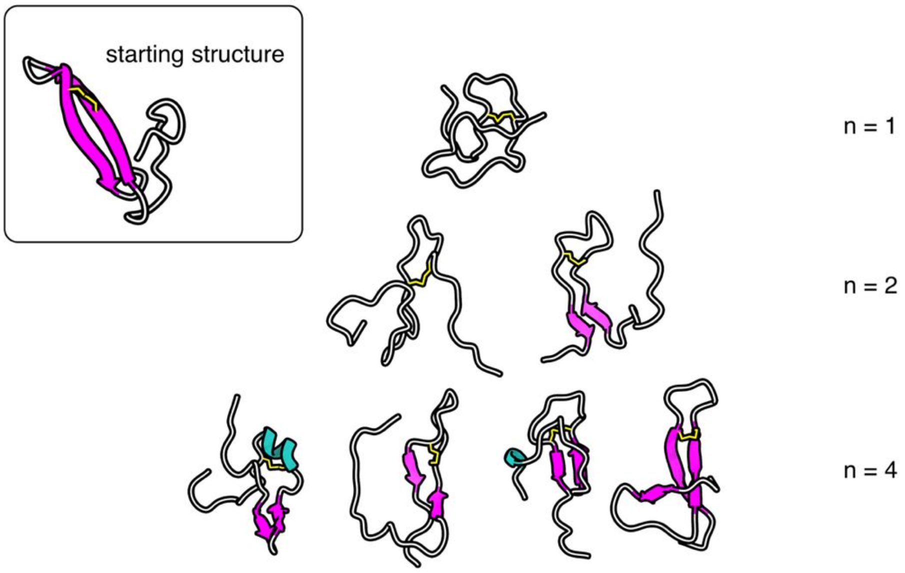

Figure S9. Structures of the monomeric units of the Aβ(1-40)$_{CC}$ in the top clusters of oligomers from MD simulations, illustrating the increase in β-sheet propensity upon oligomer growth. The starting structure for the MD simulation (derived from pdb 2otk) of the monomer is also shown for comparison. The structures are colored according to secondary structure, gray for coil, magenta for sheets and cyan for helices. The disulfide bond is shown in yellow.

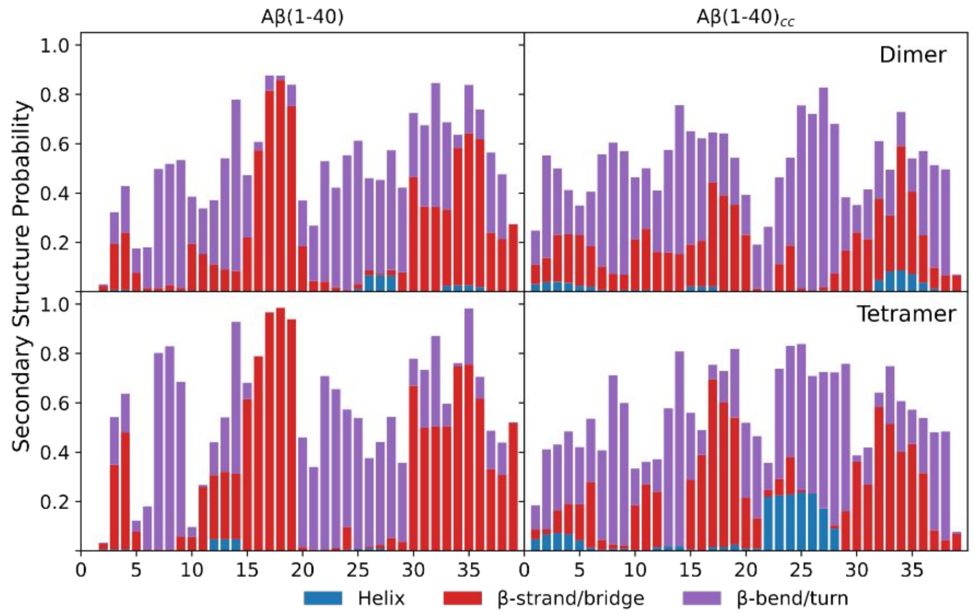

Figure S10. Probability of secondary structure of the residues of Aβ(1-40) (left) and Aβ(1-40)$_{CC}$ (right) for dimers (top) and tetramers (bottom). The bars represent the average additive secondary structure probabilities consisting of helix (blue), β-strand/bridge (red), and turn or bend (violet). The difference to 1.0 presents (shown in white) represents the random coil state.

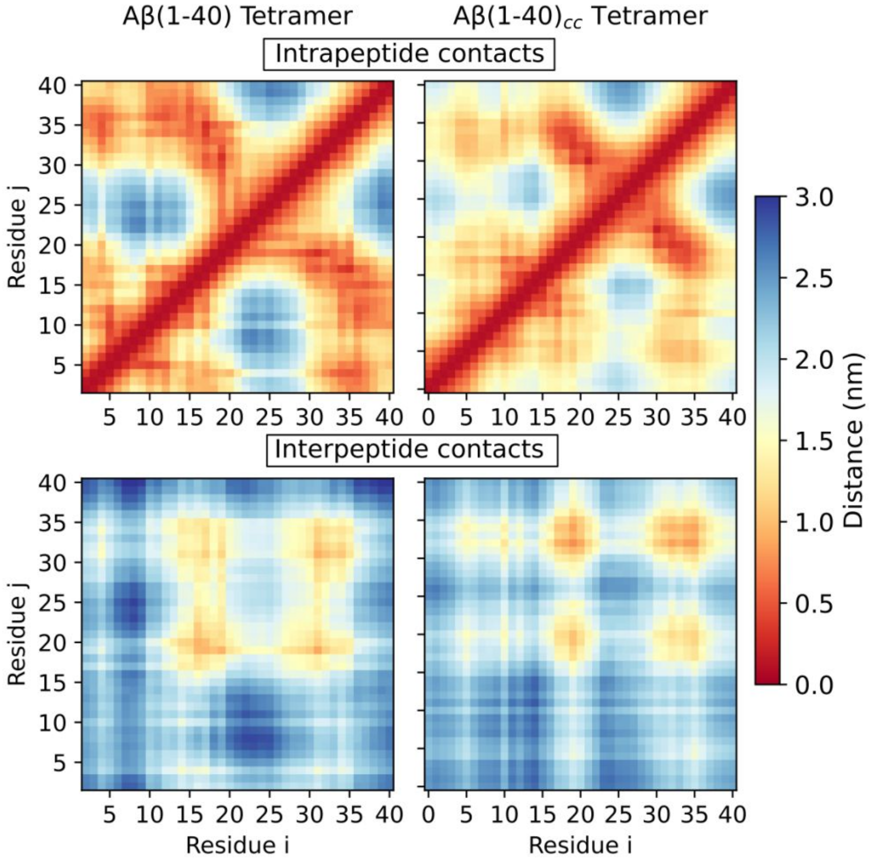

Figure S11. The average intra- (top) and interpeptide (bottom) contacts, shown as distances between residues for tetramers of Aβ(1-40) (left) and Aβ(1-40)$_{CC}$ (right), obtained from MD simulations. The intrapeptide contacts were averaged over the four peptides in the tetramers and the results are shown as symmetric matrices. For the interpeptide contacts, the distances were averaged over all possible peptide pairings. The resulting matrices are almost symmetric as in converged simulations one should obtain d(i,j') = d(i',j) where the prime indicates that the two residues i and j' belong to different peptides. The color bar on the right shows the average intra-/inter-residue distance (in nm).

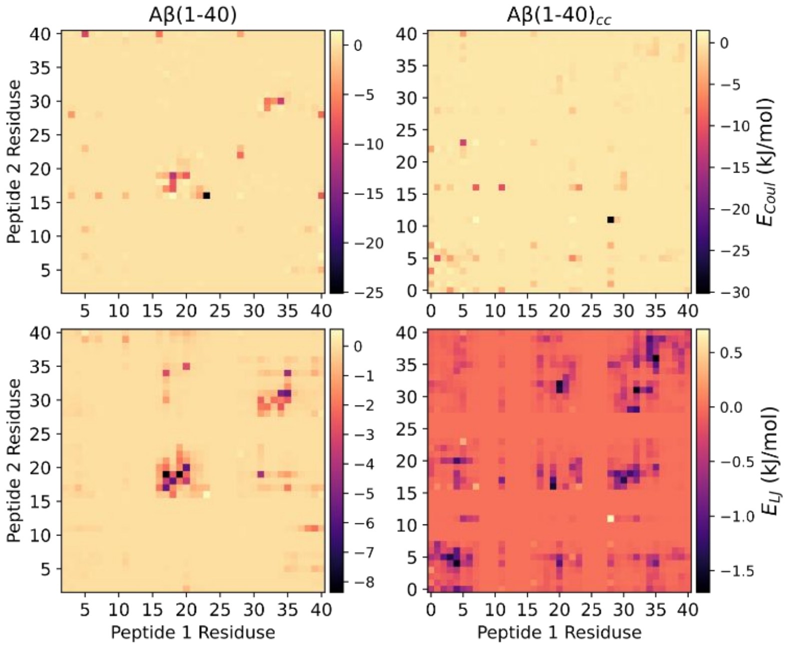

Figure S12. Average interpeptide interaction energies between residues, decomposed into Coulomb (top) and Lennard-Jones (bottom) interactions, for Aβ(1-40) (left) and Aβ(1-40)$_{CC}$ dimers (right). Please note that energies are only calculated for relevant residue numbers 1-7, 11, 16-23 and 28-40. Moreover, the energy scales in the four plots are different from each other (see color bars) in order to provide a good resolution of the energies.

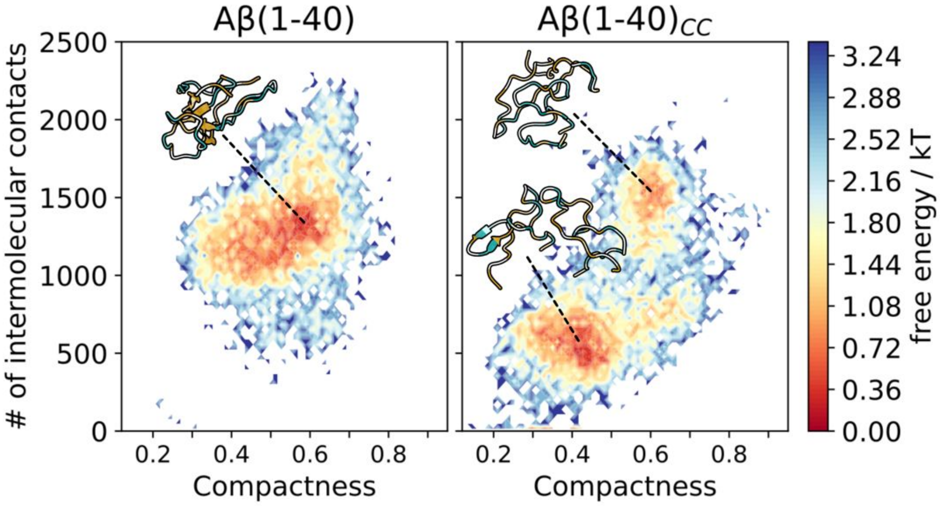

Figure S13. Cluster analysis of Aβ(1-40) and Aβ(1-40)$_{CC}$ with calculated free energy surfaces. Aβ(1-40)$_{CC}$ displays 930 clusters, whereas Aβ(1-40) has 670 clusters.

The free energy surfaces show that the formation of compact and extended structures primarily relies on intermolecular interactions. According to the free energy surface analysis of Aβ(1-40)$_{CC}$, intermolecular contacts fluctuate between low and high numbers, leading to two states of compactness centered around 0.4 and 0.6. On the other hand, Aβ(1-40) exhibits a greater number of contacts and is predominantly centered around a compactness value of 0.6.

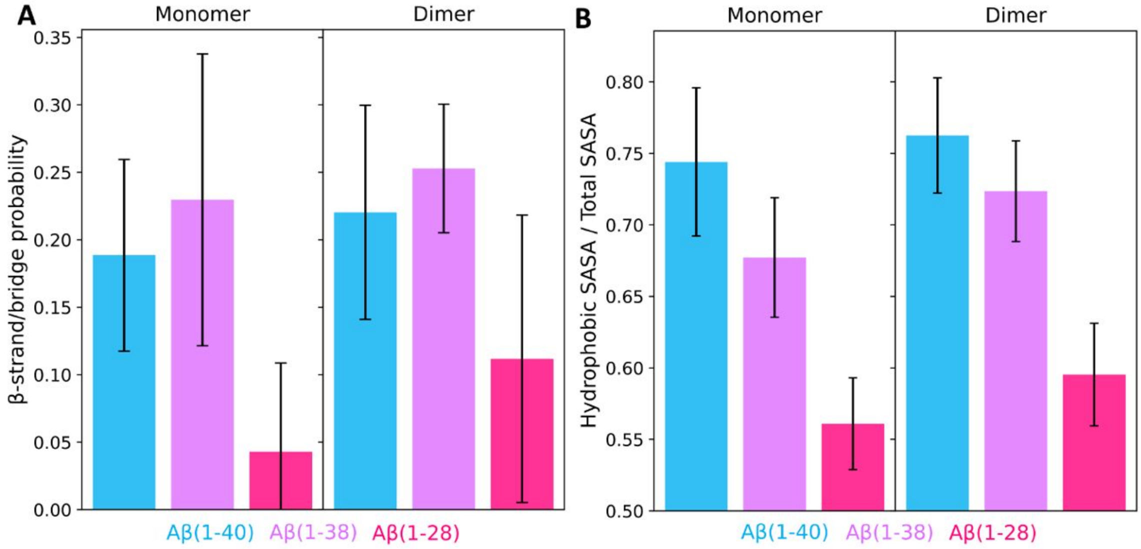

Figure S14. **A)** Average β-strand/bridge content probability for Aβ(1-40), Aβ(1-38) and Aβ(1-28) monomers and dimers. **B)** The ratio between the average hydrophobic SASA and the average total SASA for Aβ(1-40), Aβ(1-38) and Aβ(1-28) monomers and dimers.

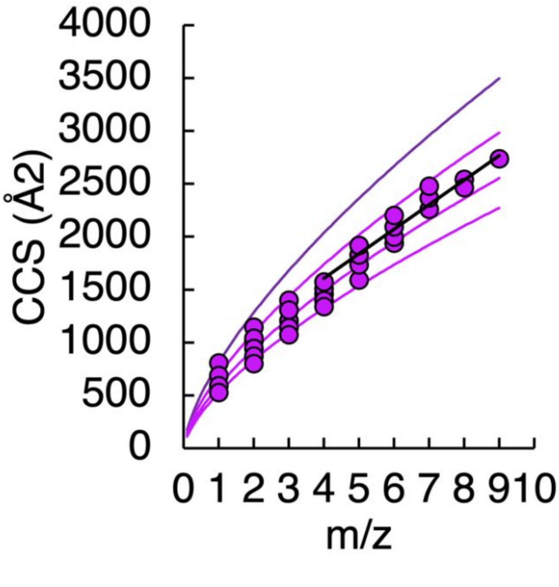

Figure S15. Measured collision cross sections (CCS) for each oligomeric state of Aβ(1-38). Solid purple lines represent the theoretical isotropic growth originating from each monomer state. A black solid line represents a linear fit to the experimental values for larger oligomers that deviate from isotropic growth.

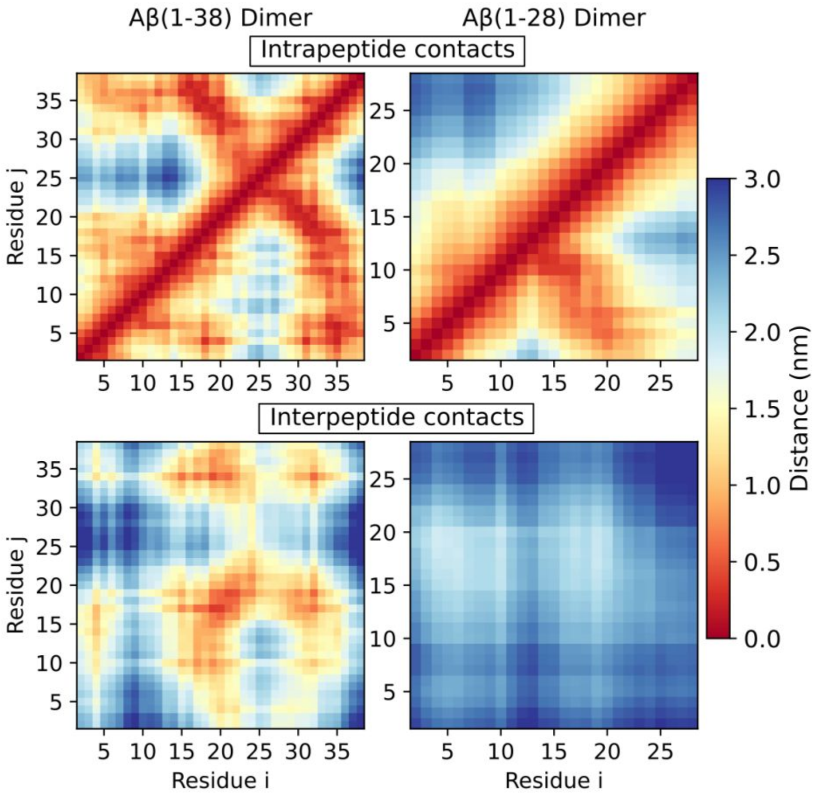

Figure S16. The average intra- (top) and interpeptide (bottom) contacts, shown as distances between residues for dimers of Aβ(1-38) (left) and Aβ(1-28) (right), as obtained from MD simulations. The intrapeptide contacts are shown per peptide composing the dimers in the upper left and lower right triangle of the maps. The interpeptide contacts are shown as distances d(i,j') where the prime indicates that the two residues i and j' belong to different peptides. The resulting matrices are almost symmetric as in converged MD simulations one should obtain d(i,j') = d(i',j). The color bar on the right shows the average intra-/inter-residue distance (in nm).

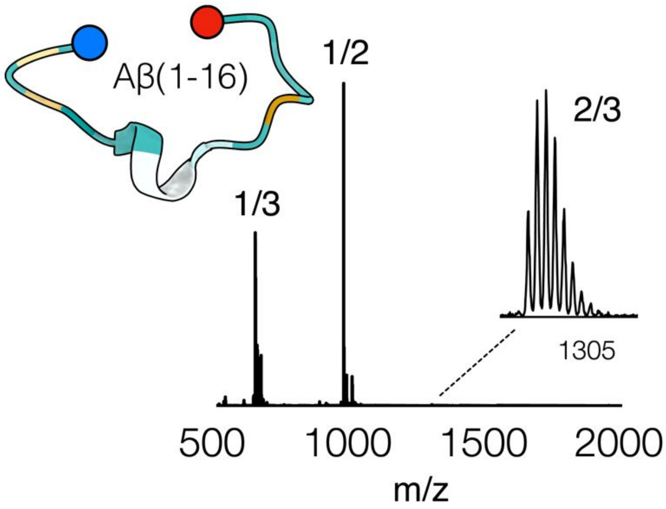

Figure S17. Mass spectrum of Aβ(1-16) with peaks annotated by their oligomeric state/charge state. The NMR structure of Aβ(1-16) (pdb 1ze7 (3)) is shown and is colored according to hydrophobicity (orange = hydrophobic, blue = hydrophilic). The N-terminus is highlighted in blue, while the C-terminus is highlighted in red.

References:

1. Cole, C., Barber, J. D., and Barton, G. J. (2008) The Jpred 3 secondary structure prediction server. *Nucleic Acids Res*. **36**, W197–W201
2. Morimoto, A., Irie, K., Murakami, K., Masuda, Y., Ohigashi, H., Nagao, M., Fukuda, H., Shimizu, T., and Shirasawa, T. (2004) Analysis of the secondary structure of β-amyloid (Aβ42) fibrils by systematic proline replacement. *Journal of Biological Chemistry*. **279**, 52781–52788
3. Zirah, S., Kozin, S. A., Mazur, A. K., Blond, A., Cheminant, M., Ségalas-Milazzo, I., Debey, P., and Rebuffat, S. (2006) Structural changes of region 1-16 of the Alzheimer disease amyloid beta-peptide upon zinc binding and in vitro aging. *J Biol Chem*. **281**, 2151–61
